# Biogeographic implication of temperature-induced plant cell wall lignification

**DOI:** 10.1038/s42003-022-03732-y

**Published:** 2022-07-29

**Authors:** Alan Crivellaro, Alma Piermattei, Jiri Dolezal, Paul Dupree, Ulf Büntgen

**Affiliations:** 1 Department of Geography, University of Cambridge, CB2 3EN Cambridge, United Kingdom; 2 Forest Biometrics Laboratory, Faculty of Forestry, Stefan cel Mare University of Suceava, 720229 Suceava, Romania; 3 Institute of Botany, Academy of Sciences of the Czech Republic, 379 01 Trebon, Czech Republic; 4 Department of Botany, Faculty of Science University of South Bohemia, 370 05 Ceske Budejovice, Czech Republic; 5 Department of Biochemistry, University of Cambridge, CB2 1QW Cambridge, United Kingdom; 6 Swiss Federal Research Institute WSL, 8903 Birmensdorf, Switzerland; 7 Global Change Research Institute CAS, 603 00 Brno, Czech Republic; 8 Department of Geography, Faculty of Science Masaryk University, 611 37 Brno, Czech Republic

**Keywords:** Plant ecology, Biogeography

## Abstract

More than 200 years after von Humboldt’s pioneering work on the treeline, our understanding of the cold distribution limit of upright plant growth is still incomplete. Here, we use wood anatomical techniques to estimate the degree of stem cell wall lignification in 1770 plant species from six continents. Contrary to the frequent belief that small plants are less lignified, we show that cell wall lignification in ‘woody’ herbs varies considerably. Although trees and shrubs always exhibit lignified cell walls in their upright stems, small plants above the treeline may contain less lignin. Our findings suggest that extremely cold growing season temperatures can reduce the ability of plants to lignify their secondary cell walls. Corroborating experimental and observational evidence, this study proposes to revisit existing theories about the thermal distribution limit of upright plant growth and to consider biochemical and biomechanical factors for explaining the global treeline position.

## Abstract (web summary)

A global survey of lignin content in plant cell walls corroborates suggestions that cold temperature limits upright tree growth.

## Introduction

Representing the potential cold distribution limit of upright tree growth^1^, elevational and latitudinal treelines are one of the most fascinating bioclimatic and biogeographic boundaries on our planet that received much scientific attention over the past centuries. Set by thermal growth constraints^2^, the potential treeline position describes the general distribution limit of the lifeform tree. This temperature-induced range limit is usually above the elevational or latitudinal limit of individual tree species that exhibit different freezing tolerances^1^. The treeline isotherm is closely related to the potential, thermal limit of wood tissue formation and increases in elevation towards the equator, reaching almost 5000 m above sea level (m asl) in southern Tibet and the Bolivian Andes^3,4^. The northernmost treeline approaches sea level at circa 73° North on the Russian Taimyr Peninsula in northern Siberia.

More than 200 years after Alexander von Humboldt’s pioneering work on the upper treeline^5^ and many fundamental studies afterwards^1–9^, our understanding of the eco-physiological, biochemical and biomechanical drivers of the tree-limiting isotherm is, however, still limited. The ability of small plants to establish, grow and age above the potential treeline position suggests a lifeform-specific constraint that affects tall trees and upright growing shrubs first^6^, followed by smaller, multi-stem and often prostrate dwarf shrubs at slightly lower thermal thresholds and finally also perennial herbs at sites with the coldest climate conditions^10,11^. It has been further reported that tree shoot development and wood cell production both cease at around 5–7 °C^1,6^, which roughly refers to a limit of water uplift and height growth^12,13^, but appears unrelated to carbon acquisition by photosynthesis^14^. Short plants that grow at higher elevations and latitudes are likely to benefit from favourable microclimatic ground-level conditions^15^. Their morphological advantage of escaping from cold free air conditions alone, however, does not offer eco-physiological, biochemical and/or biomechanical explanations for the global treeline isotherm.

**Fig. 1:**
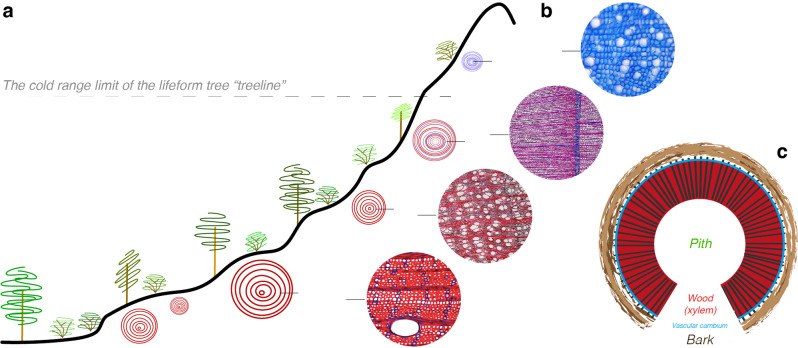
Concept of thermally constrained plant cell wall lignification. **a** Trees, shrubs, dwarf shrubs and herbs below the treeline mainly contain lignified cell walls, whereas the lifeform trees at their upper and northern distribution limit occasionally exhibit ‘Blue Rings’, and only small plants with often less lignified cell walls can grow above the treeline isotherm (the thermally induced cold distribution limit of the lifeform tree). **b** Double-stained anatomical thin sections show an almost non-lignified, five cm small *Ladakiella klimesii* from Ladakh desert in the Himalaya at around 6150 m asl, a ‘Blue Ring’ of a *Pinus uncinata* from the Spanish Pyrenees at circa 2200 m asl, a near fully lignified, 50 cm-high *Capparis spinosa* from Cyprus at 50 m asl, and an almost fully lignified, 15 m tall *Juglans regia* from northeast Italy at 60 m asl (from top to bottom). The horizontal black lines refer to the growth direction that is also indicated on the corresponding cross-sections along the elevational gradient. All photos are original and have been taken by one of the authors. (**c**) Schematic representation of a cross-section of a plant stem with secondary growth, including the wood anatomical structure of dicotyledonous angiosperms (see Fig. 2 for details).

**Fig. 2:**
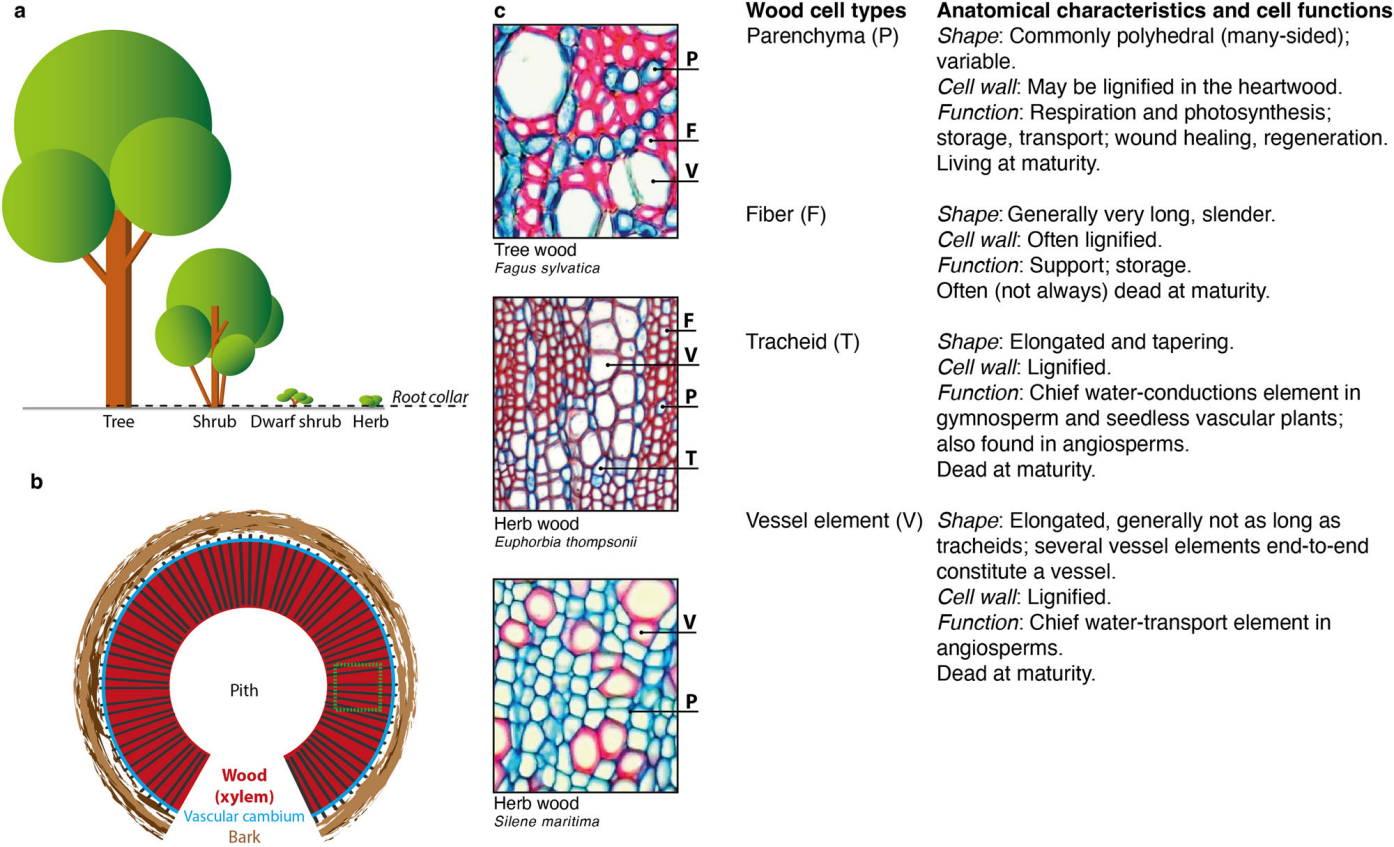
Variation in cell wall lignification. **a** Lifeforms included in this study range from trees, shrubs and dwarf shrubs to herbs. The dashed line refers to the root collar where all stem samples have been taken. **b** Cross-section of a plant stem with secondary growth represents the common wood structure of dicotyledonous angiosperms. The dashed green square on the xylem refers to the outermost mature wood from where all anatomical thin sections were taken. **c** Cross-section of plant stems with secondary growth of a tree (*Fagus sylvatica*), as well as two herbs (*Euphorbia thompsonii* and *Silene maritima*), including characteristic details of woody cell types in dicotyledonous angiosperms. All photos are original and have been taken by one of the authors.

Although the initiation, expansion and maturation of wood cells all depend on the interplay of favourable climate conditions throughout the growing season^16,17^, direct and indirect abiotic requirements for the biosynthesis of cell wall cellulose, hemicellulose and lignin compounds are not yet fully understood. While cold temperatures are known to limit the production of new cells^18^, it remains unclear if the proportion of cell wall lignin, cellulose and other polysaccharides are also thermally constraint^16^. Despite some persisting uncertainties associated with the eco-physiological process of plant cell wall lignification under open, non-laboratory, growing conditions^19^, it has been argued that lignin biosynthesis was one of the most important evolutionary achievements after the land invasion of plants around 460 million years ago^20,21^. The lignin composition in different plant species and their tissues can vary substantially (Figs. 1 and 2). Heterogeneous polymers usually fill the macro pores in-between the holocellulose structure of newly formed cell walls. Among other functions, including the defence against herbivores and fungi^22^, the hydrophobic and mechanical characteristics of lignin not only enable water uplift but also protect water-conducting cells from implosion under extreme drought stress^23^. The relative amounts of lignin, cellulose, and hemicellulose in the cell walls of plant stems enhance their mechanical strength^24^. The strengthening role of lignin is, to some degree, essential for most lifeforms, as it allows plants to grow upright, cope with mechanical stress, and transport water along vertical distances (Fig. 2).

Water-conducting stem cells of taller plants require insoluble lignin and hydrophobic compounds to stiffen and waterproof their walls to facilitate water uplift and resist strong negative pressure^25^. Since depressions in the vascular system of plants can reach up to 80 bar under very warm and dry conditions^26^, water-conducting cells are highly vulnerable to implosion if their cell walls are not strengthened by lignin. In response to mechanical demands associated with their sheer body size, self-supporting plants also rely on lignin to provide compression strength to stand upright. Lignin deposition likely ranges amongst the final steps of plant cell wall biosynthesis^27^, which establishes functionally mature cells, in most cases, just before their genetically programmed death. With up to 25% of dry weight in wood^28^, lignin is the second most abundant biomolecule on our planet (after cellulose). Although it is well accepted that lignin enhances the mechanical strength of plants and enables their intrinsic water transport, and much is known about those processes underlying lignin deposition in cell walls, its consideration and interpretation in macroecology and biomechanics is still marginal^9^. Most importantly, the role of lignin deposition in the cell walls of woody plant stems has never been studied systematically in the context of large-scale biogeographic patterns.

Here, we apply a simple but efficient, newly explored wood anatomical technique to estimate the degree of cell wall lignification (hereinafter DCWL). Based on double-stained thin sections, our new index microscopically quantifies the proportion of lignified secondary cell walls in the stems of 1770 plant species from 198 sampling sites on six continents. Motivated by recent dendrochronological, wood anatomical and plant physiological evidence of thermally constrained stem cell wall lignification^9^, we aim to address two fundamental, yet unanswered questions in treeline research^1^: “What is causing the low-temperature range limit of tree species and the range limit of the tree lifeform?” and “Why does the lifeform tree reach a cold limit beyond which numerous smaller stature, alpine, or arctic plant species are thriving?”. Based on the herein observed differences in DCWL across our large-scale plant collection from a maximum range of biogeographic habitats between the lowest sampling sites at sea level and the highest collection point at around 6150 m asl, our study hopefully contributes to further discussions and hypotheses about the complex interplay of biotic and abiotic factors that influence the global treeline position.

## Results

### Anatomical diversity of plant cell wall lignification

**Fig. 3:**
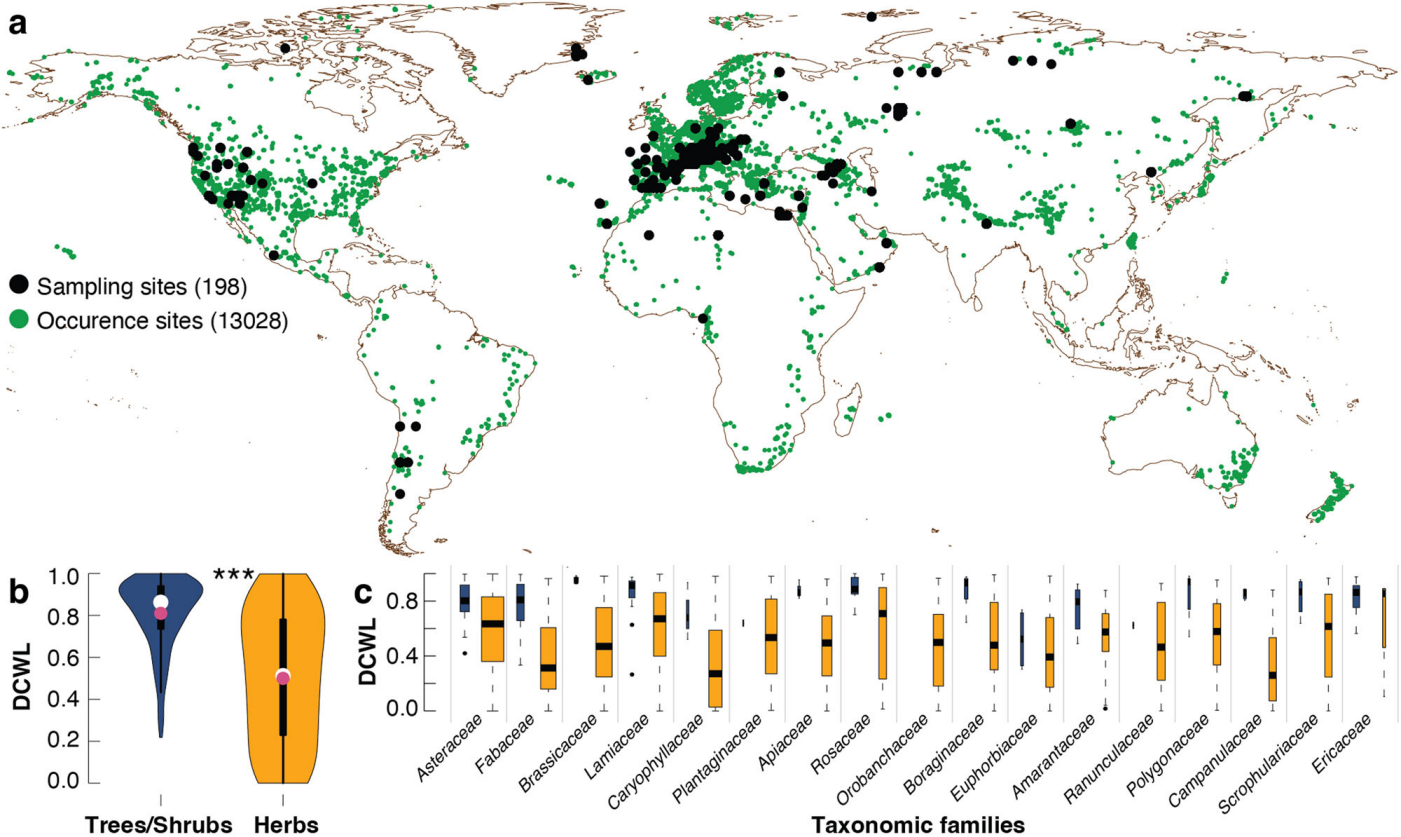
Global dataset of plant cell wall lignification. **a** Black dots show the location of the 198 sampling sites on six continents between 40°S and 80°N, and between sea level and around 6150 m asl, superimposed on corresponding species’ occurrence data (green dots) obtained from the Global Biodiversity Information Facility (GBIF)^51^. **b** Violin plots describe the distribution of DCWL in trees and shrubs (dark blue), as well as in herbs (herbaceous dicots; orange), which ranges from 0 to 1 (from almost un-lignified to almost completely lignified). The shape of the violin plot refers to the proportion of data. Black boxes within each violin show the interquartile range and whiskers the 95% confidence intervals. Red is the mean and white dot is the median of the data. Three asterisks (***) indicate significant (*p* < 0.001) differences of a paired-sample *t*-test between the mean DCWL in trees/shrubs and herbs (scores for trees and shrubs: mean = 0.81, standard deviation = 0.17 and for herbs: mean = 0.50, standard deviation = 0.31; t (25.7) = 1115.1). **c** Box and whisker plot of DCWL of the 17 plant families with more than 25 species split into trees and shrubs (dark blue), as well as herbs (orange). Box width is proportional to the number of species, box length indicates the interquartile range, whiskers the 5th and 95th percentiles, and horizontal black lines the median.

**Fig. 4:**
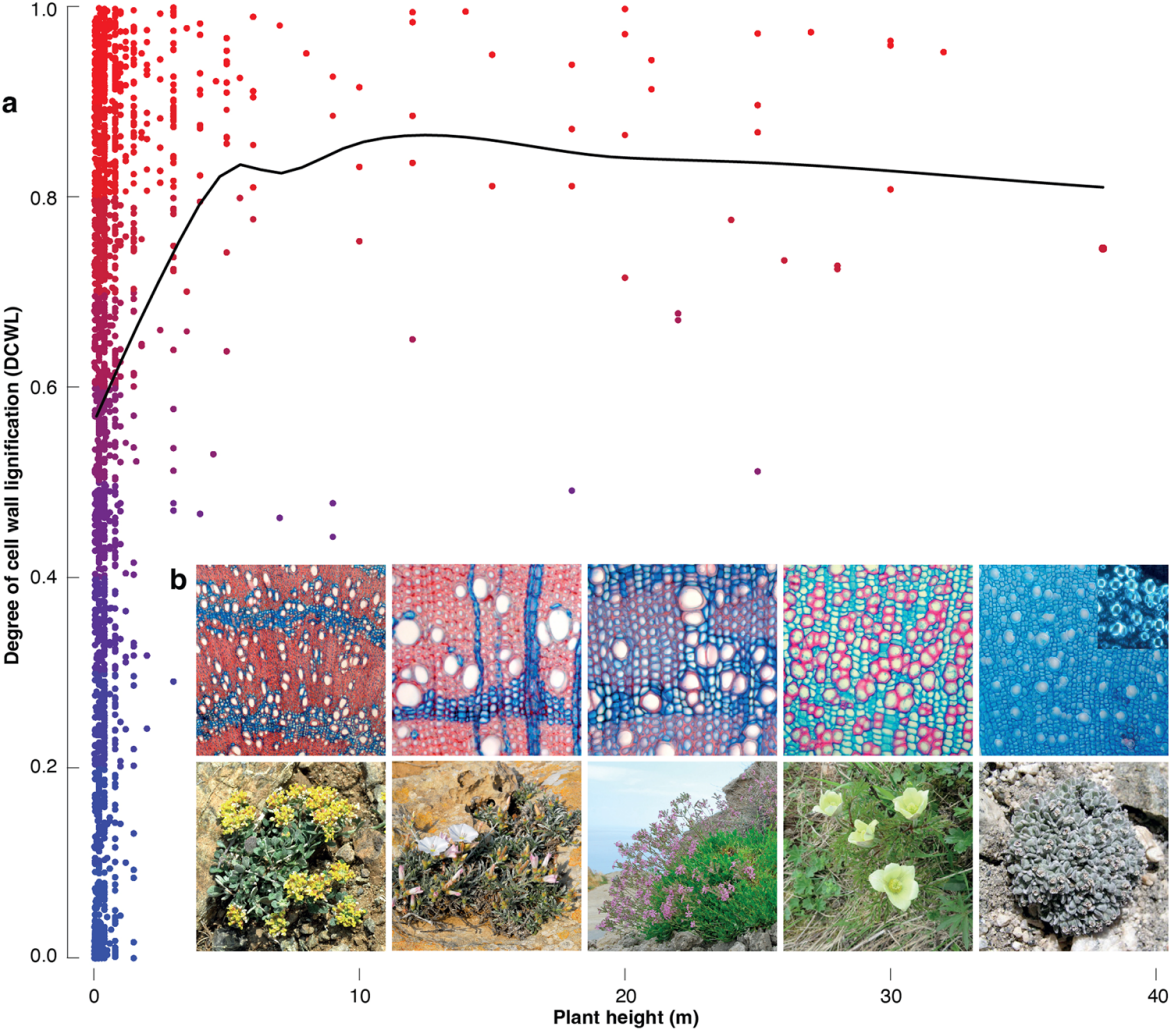
Relation between cell wall lignification and plant height. **a** Dots represent 1770 different species (i.e., one sample per species), with colours ranging from blue (less lignified) to red (lignified). The curve is a locally weighted polynomial Loess Regression that is fitted to the mean DCWL values of each 10-cm plant height class. **b** Microscopically magnified double-stained thin sections of five species of angiosperms dicotyledonous, which visualise the wide range of DCWL in herbs since Safranin and Astra Blue stain lignified and less lignified cell walls red and blue, respectively: *Alyssum akamasicum* is a perennial herb that can reach up to ten cm in vertical height and contains wood with lignified vessels, fibres, and less lignified parenchyma cells, but no rays (100×). *Convolvulus oleifolius* is a creeping shrub that can reach up to 50 cm in horizontal length and contains xylem with lignified vessels, fibres and less lignified parenchyma cells and rays (200×). *Silene fruticosa* is a perennial herb that can reach up to 15 cm in vertical height and contains xylem with lignified vessels, fibres, and less lignified parenchyma cells but no rays (200×). *Pulsatilla alpina* is a perennial herb that can reach up to 15 cm in vertical height and contains xylem with less lignified vessels and parenchyma cells, but no fibres and rays (200×). *Ladakiella klimesii* is a perennial herb that can reach up to ten cm in vertical height and contains xylem with almost non-lignified vessels and parenchyma cells, but no fibres and rays (200×). The inset figure (top right) shows a portion of the microsection under polarised light to highlight crystalline cellulose (bright) that increases cell wall mechanical properties in less-lignified vessels (200×). All photos are original and have been taken by one of the authors.

Our dataset of 1770 woody plant species, supplemented by the precise information on latitude, longitude, elevation, temperature and precipitation of 13,028 species-site combinations (Methods), resembles the global distribution of species-specific differences in microscopically assessed DCWL (Fig. 3a). DCWL is a dimensionless index of the fraction of lignified cell wall area relative to the total cell wall area, which ranges from ‘0’ in almost completely blue stained (near un-lignified) anatomical images to ‘1’ in almost completely red (near fully lignified) images. Our approach allows the extent of cell wall lignification to be estimated over undisturbed areas of stem cross-sections that include all different cell and tissue types, such as fibres, parenchyma, and vessels. While upright growing stems of trees and shrubs always exhibit some DCWL (Fig. 3b), the stems of much smaller herbs range from nearly un-lignified to almost fully lignified. Evidence for higher DCWL in trees and shrubs, and a much more variable DCWL in herbs, is further exhibited by each of the major plant families (Fig. 3c). Though herbs with low DCWL are common, DCWL increases with plant height, and trees taller than circa ten metres always have an estimated DCWL above 0.5 (Fig. 4a). Most importantly, poorly lignified trees and upright growing shrubs cannot exist, because their self-supporting stems must be able to cope with static and dynamic stressors, as well as long-distance root-to-leaf water transport.

The herein observed variability of DCWL in the stem sections of herbs contradicts the common belief that small plants are always poorly lignified. This finding also challenges the misperception that herbs are non-woody plants. Although one would expect smaller plants to experience less mechanical strength and thus to require less lignified cell walls, this size-driven assumption is not always true. Different herb species can adapt to different environmental conditions^29,30^, and the various roles lignin play for their wood, such as resisting negative pressure in the vascular system in dry areas, is reflected by the observed DCWL variability in small plants. DCWL depends on both, the anatomical proportion and arrangement of different wood cell types, as well as their cell wall chemical composition, which may differ within and between plants (Figs. 1 and 2). Starch-storing parenchyma cell walls always stain blue in the sapwood, while DCWL may vary considerably between fibre cells and conducting vessels that provide structural and hydraulic functions, respectively. The wood anatomical diversity of our dataset includes a wide range of plant species and site conditions (Figs. 3 and 4), such as the fibreless wood of some small herbs, in which the mechanical function is relocated to thick-walled vessels (e.g., *Pulsatilla*), or the heavily lignified and thick-walled fibre-rich wood of other herbs (e.g. *Convolvulus*) (Fig. 4b).

### Geographical diversity of plant cell wall lignification

**Fig. 5:**
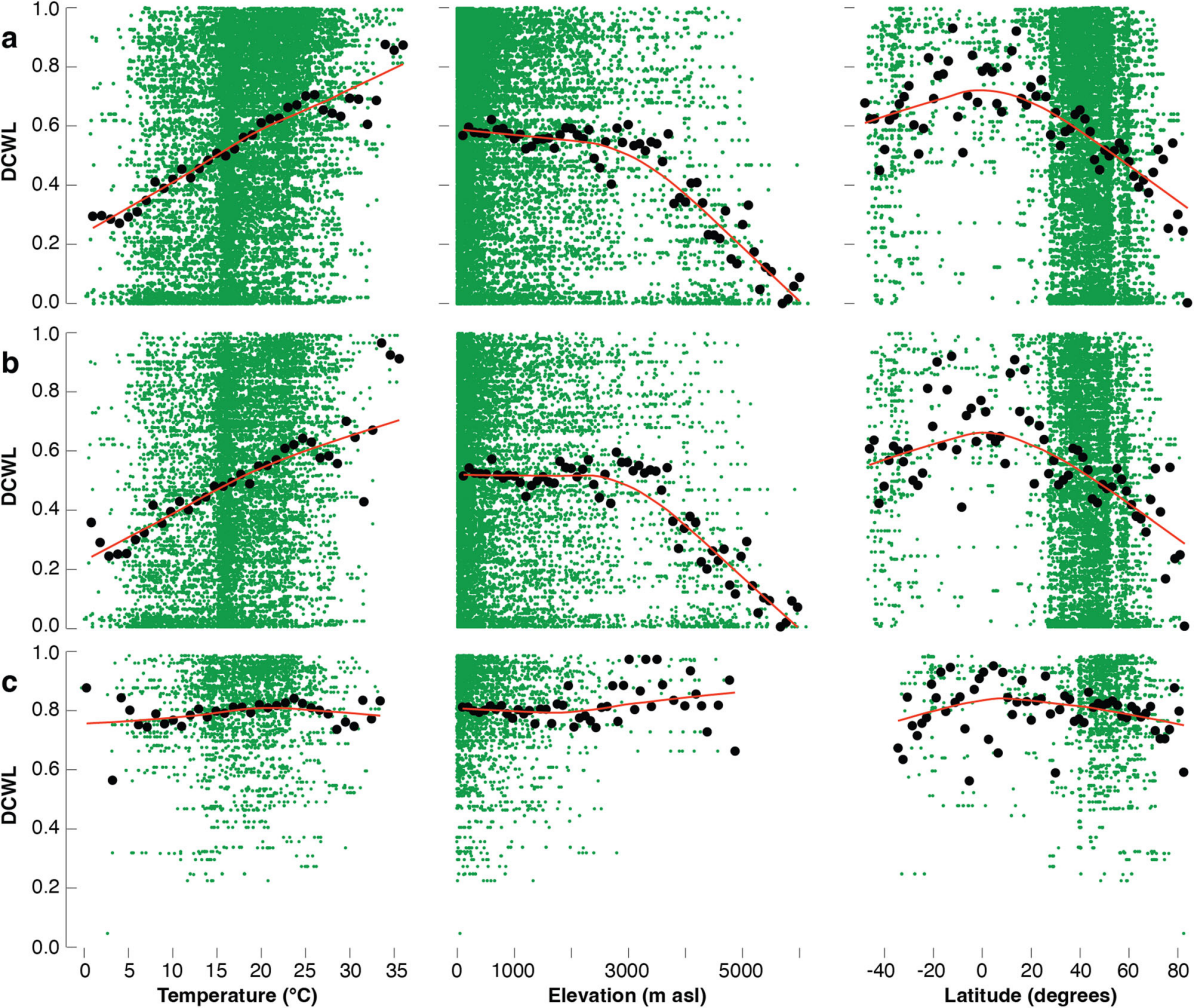
The influence of temperature, elevation and latitude on plant cell wall lignification. The role temperature, elevation and latitude play for DCWL in **a** all species, **b** herbs and **c** trees and shrubs (black dots), with the red lines being locally weighted polynomial Loess Regressions fitted to mean DCWL values. Green dots represent species-site combinations, while black dots are mean values of DCWL computed in each class of 1 °C temperature of the warmest quarter, of 100 m elevation, and of 2° latitude. Similar results are obtained when plotting the data separately for specimens collected in natural growing conditions, in botanic gardens, and for herbs smaller than 10 cm in vertical height (Supplementary Fig. 3).

**Fig. 6:**
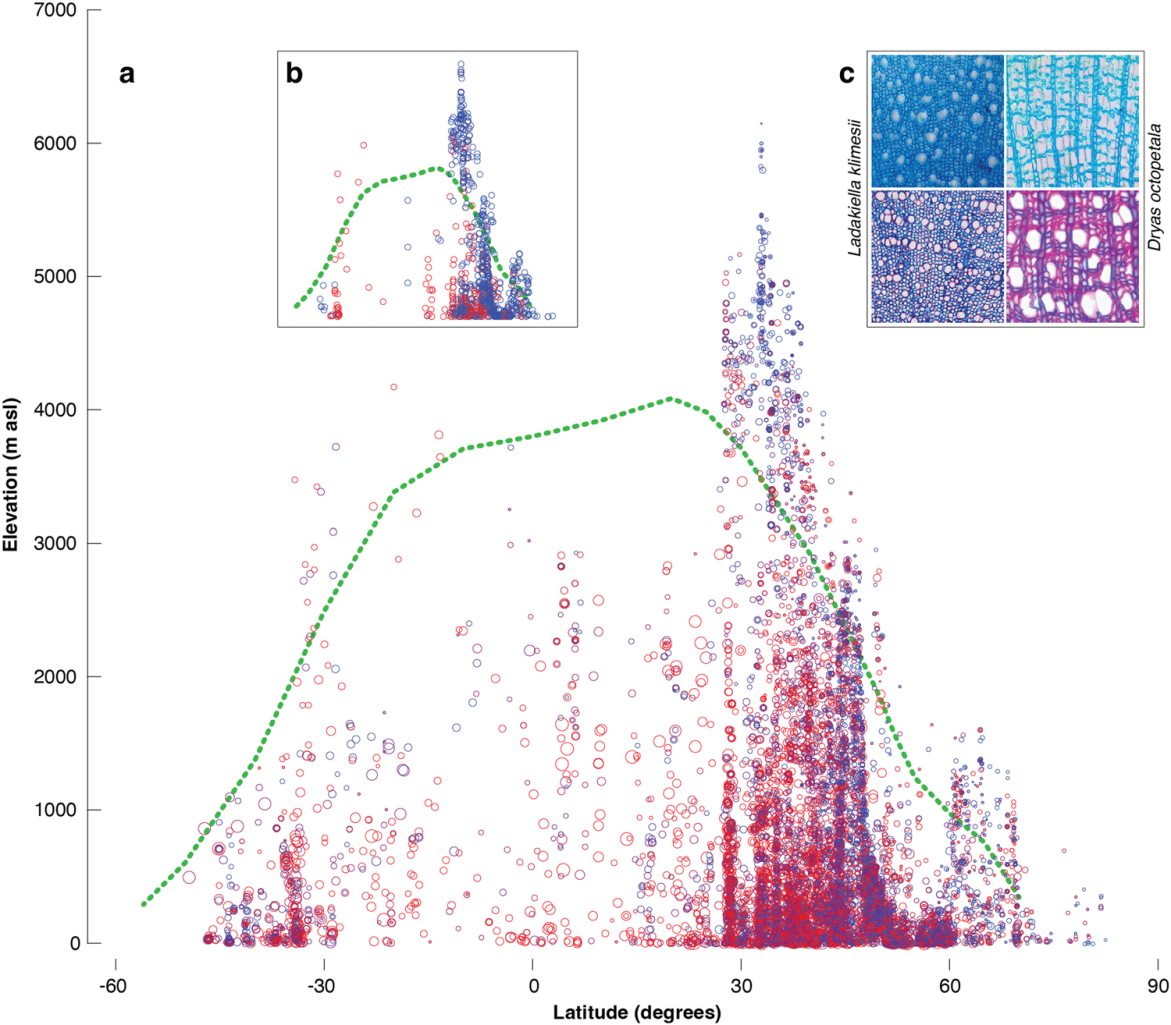
Global distribution of plant cell wall lignification. **a** Elevational and latitudinal distribution of DCWL that ranges from less lignified (blue circles) to more lignified (red circles). Circle sizes refer to vertical plant heights from two centimetres and circa 40 m (small to large). The green line roughly resembles the temperature-induced global treeline position^1,46^. **b** The same plot but limited to the 2nd and 98th DCWL percentiles (see Supplementary Fig. 4 for lifeform differences). **c** Double-stained stem cross-section (top left) of a four cm small *Ladakiella klimesii* that was growing for more than 20 years under very harsh conditions at around 6150 m asl in the Ladakh desert, Himalaya^11^. The stem of this plant reveals overall less lignified cell walls, whereas other individuals of the same species that were growing at lower elevations show more lignified wood vessels (bottom left). The same pattern is evident for *Dryas octopetala* from a high-latitude site in coastal east Greenland, showing less lignified wood (top right) compared to samples of the same species growing in warmer conditions in the European Alps (bottom right). All photos are original and have been taken by one of the authors.

DCWL in terrestrial plants is generally lower at sites with cold growing season temperatures (Fig. 5a), which are usually located towards higher elevations and higher latitudes. This pattern is most pronounced in herbs but not in trees and shrubs that were not found at the network’s climatic extremes (Fig. 5b, c). While similar results have been reported for gymnosperm wood at much smaller spatial scales^31^, systematic large-scale evidence has been missing so far. To account for the potential effect of an increasing proportion of herbs towards colder sites, we analysed the relative contribution of elevation, latitude, temperature, precipitation, and plant height for DCWL (Supplementary Table 1). Correlations between DCWL and the climatic variables were quantified for all species and each lifeform separately (Supplementary Table 2). Each variable significantly affected DCWL (except for elevation, where the net effect is only marginally significant). The strongest effect was found for temperature. No relationship was found between phylogeny and the elevation and latitude of our samples because those plant families growing at extreme high-elevation and high-latitude sites (e.g. *Asteraceae*, *Brassicaceae*, *Saxifragaceae* and *Caryophyllaceae*) also include individuals from less extreme environments (Supplementary Fig. 1). When species occurrences are plotted against annual precipitation totals and mean growing season temperatures (Supplementary Fig. 2), less lignified, small plants are predominantly found in colder climates, especially where snow cover persists for most of the year. In contrast, more lignified herbs, shrubs, and trees are widespread across warmer climates, with overall higher DCWL at lower elevations and lower latitudes where growth conditions are favourable (Fig. 5). The relationships between DCWL and temperature, elevation and latitude are confirmed by subsets from natural habitats, all samples from botanic gardens, and all herbs of smaller ten cm vertical height (Supplementary Fig. 3). Our results imply a generalised picture of temperature-induced DCWL distribution supportive of a new theory about the global treeline position (Fig. 6). Under warmer climates at lower elevations and lower latitudes, the stem cell walls of trees and shrubs are always lignified. However, the stem cell walls of herbs are generally less lignified at high elevations and high latitudes, which is particularly the case when herbs exceed the potential treeline position (Supplementary Fig. 4).

## Discussion

Observations of different plant species at different sites provide evidence for a universal thermal threshold of stem cell wall lignification across angiosperm and gymnosperm lineages. Extremely cold conditions during the growing season, under which the biosynthesis and deposition of lignin in the cellulosic matrix of newly formed cell walls might be hindered^32^, may therefore contribute to the upper elevational and latitudinal distribution limit of upright tree and shrub growth.

Seedlings of seven broadleaf tree species that were growing under experimental conditions at different soil temperatures showed that roots formed below 7 °C had reduced mechanical strength because of limited lignification^33,34^. It has been further shown that the freezing tolerance and cold acclimation of *Arabidopsis thaliana* (L.) Heynh can be enhanced when the lignin cell wall content was reduced^35^. Moreover, independent evidence for a thermally induced lack of cell wall lignification emerges from recent advances at the interface of dendrochronology and quantitative wood anatomy^9,36^. So-called ‘Blue Rings’ describe a wood anatomical feature most likely triggered by the occurrence of ephemeral cold spells during the growing season^7,37,38^. ‘Blue Rings’ have been observed in different conifer species at sites where summer temperature conditions predominantly control radial stem growth. ‘Blue Rings’ denote layers of less lignified (blue) latewood cells that generally coincide with abrupt summer cooling^7,37^, often following large volcanic eruptions (Supplementary Fig. 5). A study on the phenology and physiology of the Snowbell *Soldanella pusilla* (*Primulaceae*) at around 2450 m asl in the central Swiss Alps has further reported temperature constraints on cell wall lignification of flower-bearing shoots^8^. Surviving below the snowpack during winter months at circa 0 °C, the herb’s flower stalk only lignifies when exposed to warmer temperatures after snowmelt, enabling flowers to grow 60 mm upright above the ground^8^.

Our investigation of DCWL in small plants reveals a tendency for almost entirely un-lignified cell walls at exceptionally cold sites and more lignified cell walls when growing under warmer conditions (Figs. 5 and 6). Plant growth at very low temperatures is therefore restricted to a few centimetres in vertical height, in which a short root-to-leaf distance allows sufficient sap flow in even less lignified conduits. Small alpine and arctic herbs are less exposed to ambient air temperatures^14,15^, which are often colder than surface microclimates under strong solar radiation. Though it might be argued that small plants in cold environments do not require lignified stems due to their small architecture, many short plants in warm climates contain a high degree of lignin in their secondary cell walls. In line with recent dendrochronological, wood anatomical and plant physiological evidence^9^, our study suggests the distribution limit of upright growing, tall plants towards generally colder summer mean temperatures could be related to a lack of lignin deposition in the secondary cell walls. Since our data imply a low-temperature threshold of plant cell wall lignification to be an important factor for upright growth, neither source nor sink limitations should be considered relevant for plant growth under exceptionally cold temperatures. The available plant resources (source) allow for the formation and development of new wood cells even at particularly low temperatures (sink), but sufficient cell wall lignification possibly becomes the biochemical bottleneck for the completion of the full cycle of cell development stages. Very low temperatures at the cold margin of plant growth should therefore be considered as an important factor for the disruption of the lignin biosynthesis pathway, which can result in less lignified cell walls. The global treeline, therefore, represents a sharp isotherm, where both the mechanical and hydraulic properties of wood cell walls change rapidly. Trees with sufficient DCWL to grow upright depend on a combination of long and warm enough growing season conditions^1,6^. While wood in trees at the treeline and below must, by definition, be lignified, we assume that tree seedlings well above the treeline are characterised by a reduced lignin deposition in their cell walls. Such seedlings, however, will never grow tall. For more insights into the cell biology of lignin biosynthesis, we refer to a wide range of literature, which describes the current understanding of the complex biochemical pathway involving the synthesis of monomers in the cytoplasm and their transport across the cell membrane and their polymerisation in the cell wall^39–41^. Moreover, it should be noted that different levels and forms of lignin and their role in cell wall formation are still under discussion^42,43^ and that hydraulic limitations of vertical plant growth at the upper treeline have been introduced as an extension of the ‘sink limitation’ hypothesis^6,44^. The possibility of temperature-driven changes in DCWL, however, has not been considered systematically in any previous work.

To gain deeper insights into the importance of microclimate in determining DCWL, including the biotic and abiotic causes and consequences of different forms of lignin and their role in the xylogenetic process, future research should explore the thermal limits of biosynthesis in different cell wall components, either in greenhouse experiments or by relocating plants from cold to warm and warm to cold sites^38^. Such studies, including plant stem anatomical analyses across the largest trees and smallest herbs, should also quantify the environmental controls of DCWL within and between species, as well as considering similar and different cell functional types, such as fibres, vessels, and parenchyma fractions. To further investigate the biochemical basis for a putative temperature sensitivity of lignin formation and to explore the fluidity of lignin in permeating cell walls, future research could use mutants of the model plant *Arabidopsis thaliana* (L.) Heynh, in which the expression of key enzymes required for the synthesis of lignin has been removed or enhanced. Moreover, the genetic control of different types of lignin in plant cell walls^45^ will provide new insights into the potential bias of Safranin and Astra Blue double-staining to differentiate visually between cellulose and lignin^46^. Longer and better replicated ‘Blue Ring’ chronologies from different species and locations around the world will further help us to better understand the effects of (volcanic-induced) rapid cooling on cell wall lignification^37^. In return, enhanced knowledge of the environmental drivers of plant lignin biosynthesis and lignin deposition will promote the re-assessment of treeline positions^47^, as well as the improvement of eco-physiological and global vegetation models, which would increase awareness about the effects of climate change on both, cell wall lignification and global plant distribution.

## Methods

### Global network

We quantify the relative degree of stem cell wall lignification (hereinafter DCWL) in 1770 different woody plant species from 198 disjunct sampling sites (Fig. 3a). Species collection was carried out over the last 30 years from natural and undisturbed sites, as well as from botanic gardens and cultivated lands, mostly across the Northern Hemisphere. At each sampling site, we selected adult individuals that appeared undamaged and healthy. During harvest, we sampled one adult individual per species that was usually the tallest individual of that species at that site and then recorded the plant height of that individual, as well as elevation and coordinates of the sites (from a GPS device) and sites name (from the nearest geographic feature). Small plants were dug out from the ground to expose the root collar, whereas for shrubs and trees, a stem disk was cut with a saw, or a wood core was extracted with an increment borer from the upright stem (only from the main stem in multi-stem shrubs) to avoid tension wood. The sampled portions of stem and cores were stored in sealed plastic bags to which we added several drops of 40% ethanol and kept it at 3–4 °C until the samples were sectioned. We defined the plant lifeform as a functional type, independently from any taxonomic unit. While all trees and shrubs are self-supporting, herbs comprise herbaceous flowering plants other than grasses^48^. All terrestrial plants in our dataset are dicotyledonous angiosperms capable of secondary growth that were collected on six continents either in their natural habitats or in botanic gardens. Considering only samples from one mature individual per species, the vertical plant heights of our collection range from 2 cm to around 40 m, and the specimens vary in age from annual plants to several decades^49^. Though weighted towards Europe and North America, our sampling sites cover the extreme margins of plant growth at around 6,150 m asl in the Himalayan Ladakh^11^, as well as in coastal east Greenland circa 73° North^10^. A large *Saxifraga oppositifolia* L. cushion plant from circa 4500 m asl in the central Swiss Alps most likely represents one of the coldest sites of the collection^50^.

### Spatial extrapolation

Our dataset is dominated by herbs (78.7%), followed by trees (16.9%) and shrubs (4.4%). We used the Global Biodiversity Information Facility (GBIF)^51^ to extract actual distribution coordinates of the 1770 plant species collected and analysed (Fig. 3a). Species-specific geographical information was based on occurrence data available from GBIF. A maximum of ten occurrence data points was downloaded for each species via the R package ‘spocc’ software^52^. Bioclimatic attributes and elevation data were downloaded from the WorldClim Global Climate Data^53^ at 2.5 minutes resolution. For each species occurrence data point, 12 BIOCLIM variables, representing temperature and total annual precipitation attributes of a given locality, as well as elevation, were extracted using the R package ‘raster’ software^54^. For each occurrence point, we estimated elevation, as well as annual and seasonal temperature means and precipitation totals. Covering all climatic zones and terrestrial biomes, the resulting GBIF network of 13,028 species-specific locations is denser in western Eurasia and North America compared to all other continents. The 1770 woody plant species from 118 families were classified into two main lifeforms: (i) trees, shrubs and dwarf shrubs, as well as (ii) herbs that comprise herbaceous flowering plants with vascular cambium. Ferns, grasses and graminoids, as well as climbers, stem succulents, epiphytes and aquatic plants, were not included in this study.

### Anatomical assessment

Wood samples from the outermost mature and functional stem tissues (Figs. 1 and 2), close to the root collar, were prepared for wood anatomical analyses. Stem sections for anatomical observation were cut to include fully developed secondary wood and the outermost complete growth ring, though excluding the cambial region and dead cells of the heartwood. Samples enable the estimation of DCWL free from ongoing xylogenesis processes and only represent the condition of the physiologically active xylem tissues. Thin sections were bleached with sodium/potassium hypochlorite to decolour cell walls and remove undesired cell content. Thin sections were double-stained for 3–5 min with a 1:1 blend of freshly prepared Safranin (1% in water) and Astra Blue (0.5% in water with 0.2% acetic acid), which is known to stain lignified cell walls red and less-lignified cell walls blue^55,56^. Although the absolute changes in lignin content that are visualised by the double-staining method remain unknown, there is evidence for qualitative similarities between double-staining, autofluorescence imaging and Raman spectroscopy^37,58^. In contrast to polarised light microscopy that does not visualise lignin, the staining with Safranin and Astra Blue can reveal different degrees of lignin content in wood cell walls^57^. Thin sections were washed with water and dehydrated with ethanol at increasing concentration, finally embedded in Canada balsam under the cover glass. Permanent thin sections were dried for 12–24 h at 60 °C. One microscopic image of the outermost wood, excluding still developing and not yet functional cells near the cambium and dead cells of the heartwood (Fig. 2), was digitised at 100x or 200x magnification under a transmitted light optical microscope. White areas reflect cell lumina and intercellular spaces, whereas the red parts indicate more, and the blue parts indicate less lignified cell walls, respectively. A dimensionless area index of DCWL was calculated to quantify the fraction of lignified (red-stained) cell wall area relative to the total cell wall area. DCWL ranges from ‘0’ in almost completely blue stained (near un-lignified) anatomical images to ‘1’ for almost completely red (near fully lignified) images. Our staining technique captures the lignin and holocellulose components of the secondary cell walls of all cell types in each wood sample^47^. Standard wood anatomical features of each species were previously described^58–60^. Sections were imaged using a digital camera mounted on a Nikon Eclipse E400 compound microscope, equipped with an Olympus C5050 camera. Before picturing the wood sections, we adjusted the white balance on the camera to avoid variations in colour hue from picture to picture. Magnification enabled a representation of the general xylem features for the cross-section of the species and excluded cracks or artefacts on the slide, such as transverse (end) walls in axial parenchyma cells as visible in the cross-section.

### Colour coding

In each anatomical picture, the area occupied by cell lumina and intercellular spaces (white), as well as the area occupied by lignified (red) and less lignified (blue) cell walls, were estimated with the image-analysis programme ImageJ^61^. The colour area in a picture was measured using the macro colour analysis setting colour thresholds for hue, saturation, and brightness of each colour range (red, blue and white) (Supplementary Table 3). The three colour ranges capture the variability within each colour (e.g., 'red' capture all shades from light to dark red) while ensuring a clear separation from the other two. Colour settings were defined by direct observation below the microscope and comparison with image analyses output. To calculate the degree of cell wall lignification (DCWL), we divided the lignified cell wall area (red) by the total cell wall area (red and blue): DCWL = (Lignified cell wall area (red))/(Total cell wall area (red and blue)). The degree of cell wall lignification (DCWL) distribution was graphically visualised within each lifeform and geographically visualised on an elevation vs latitude plot. Relationships between DCWL, plant height and temperature of the warmest quarter, elevation and latitude were graphically represented. We investigated the relative importance of abiotic (elevation, latitude, temperature of the warmest quarter) and biotic (plant height) control of DCWL variability in trees, shrubs, and herbs. Relative abundance data of trees and herbs were subjected to variance partitioning using partial Redundancy Analyses. This analysis was performed to overcome the problem associated that rare species (e.g., trees beyond treeline) might have a much larger influence on the analysis than more common species. Three quantitative environmental variables extracted for each occurrence point-species combination (elevation, latitude, temperature of the warmest quarter) and plant height measured from the collected plant were used as explanatory variables. The significance of the variables was tested with a Monte-Carlo permutation test performed in CANOCO 5.0 software^62,63^ (Supplementary Table 1). All tests were done using 999 permutations and a significance threshold of *p* < 0.05. Both the dependent and explanatory variables were mean-cantered and variance-scaled prior to the analyses. For data visualisation, we plotted DCWL vs elevation, latitude, and temperature of the warmest quarter with each of the analysed variables divided into classes (ten cm plant height, 1° Celsius degree temperature, 100 metres elevation, and 2° latitude) and a mean DCWL value was computed in each class. Loess Regression represents a locally weighted polynomial curve fitting the mean values of each variable and visualised the trends. Pearson’s correlation coefficients between DCWL and the individual bioclimatic variables were quantified for all species and for each lifeform separately (Supplementary Table 2).

### Reporting summary

Further information on research design is available in the Nature Research Reporting Summary linked to this article.

## Supplementary information

Supplementary Information

Description of Additional Supplementary Files

Supplementary Data 1

Supplementary Code 1

Reporting Summary

## Data Availability

All relevant data are included in the online supplementary (Supplementary Data 1).

## Code availability

All relevant codes are included in the online supplementary (Supplementary Code 1).
