# Author Correction: Biogeographic implication of temperature-induced plant cell wall lignification

**DOI:** 10.1038/s42003-023-04775-5

**Published:** 2023-04-13

**Authors:** Alan Crivellaro, Alma Piermattei, Jiri Dolezal, Paul Dupree, Ulf Büntgen

**Affiliations:** 1 Department of Geography, University of Cambridge, CB2 3EN Cambridge, United Kingdom; 2 Forest Biometrics Laboratory, Faculty of Forestry, Stefan cel Mare University of Suceava, 720229 Suceava, Romania; 3 Institute of Botany, Academy of Sciences of the Czech Republic, 379 01 Trebon, Czech Republic; 4 Department of Botany, Faculty of Science University of South Bohemia, 370 05 Ceske Budejovice, Czech Republic; 5 Department of Biochemistry, University of Cambridge, CB2 1QW Cambridge, United Kingdom; 6 Swiss Federal Research Institute WSL, 8903 Birmensdorf, Switzerland; 7 Global Change Research Institute CAS, 603 00 Brno, Czech Republic; 8 Department of Geography, Faculty of Science Masaryk University, 611 37 Brno, Czech Republic

**Keywords:** Plant ecology, Biogeography

Correction to: *Communications Biology* 10.1038/s42003-022-03732-y, published online 29 July 2022.

In the original version of the Article, the second paragraph of the Methods incorrectly stated, “Bioclimatic attributes and elevation data were downloaded from the WorldClim Global Climate Data^53^ at 2.5 degrees resolution.”

The text should read, “Bioclimatic attributes and elevation data were downloaded from the WorldClim Global Climate Data^53^ at 2.5 minutes resolution.”

The error has been corrected in both the PDF and HTML versions of the Article.

