# Supplementary material for: Biogeographic implication of temperature-induced plant cell wall lignification
Source: Commun Biol. 2022 Jul 29;5:767. doi: 10.1038/s42003-022-03732-y (PMC9338036; doi:10.1038/s42003-022-03732-y)
Supplement: Supplementary file 1 — Supplementary Information [file 42003_2022_3732_MOESM1_ESM.pdf]

Supplementary information for

Biogeographic implication of a thermally-constraint global distribution of plant cell wall lignification

by Alan Crivellaro, Alma Piermattei, Jiri Dolezal, Paul Dupree and Ulf Büntgen

Supplementary Table 1 Marginal and conditional effects of plant height, elevation, and mean temperature of warmest quarter on DCWL tested by Eigenvalues, F values and *p* values which were obtained from the redundancy analysis (RDA).

Variable	Adjusted explained variation (%)	Eigenvalues of axis 1	Eigenvalues of axis 2	Pseudo-F	P
Conditional effect of variable (constrained RDA)					
Plant height	3.53	0.0354	0.9646	478	P=0.001
Elevation	2.41	0.0242	0.9758	323	P=0.001
Mean Temperature of Warmest Quarter	8.36	0.0836	0.9164	1188	P=0.001
Latitude	2.25	0.0226	0.9774	301	P=0.001
Marginal (unique) effect of variable after accounting for the others (partial RDA)					
Plant height	2.69	0.0246	0.8874	316	P=0.001
Elevation	0.02	0.0003	0.8874	3.7	P=0.056
Mean Temperature of Warmest Quarter	3.77	0.0348	0.8874	511	P=0.001
Latitude	0.31	0.0029	0.8874	41.9	P=0.001

Supplementary Table 2 Relationships between the Degree of Cell Wall Lignification (DCWL) and bioclimatic variables. Bivariate Pearson's correlations between the Degree of Cell Wall Lignification (DCWL) and individual bioclimate variables for 1,770 species grouped by lifeform (trees and shrubs, as well as herbs). The statistical significance of the Pearson's correlation coefficients (r) is indicated as: *** *p* <0.001; ** *p* <0.01; * *p* <0.05; n.s. *p* >0.05.

		**All**		**Trees / Shrubs**		**Herbs**	
**Bioclimatic variables**		r	p-value	r	p-value	r	p-value
BIO1	Annual Mean Temperature	0.29	***	0.04	n.s.	0.25	***
BIO2	Mean Diurnal Range (Mean of monthly (max temp - min temp))	0.08	***	0.03	n.s.	0.07	***
BIO3	Iso Thermality (BIO2/BIO7) (x 100)	0.20	***	0.02	n.s.	0.17	***
BIO4	Temperature Seasonality (standard deviation x100)	-0.14	***	0.03	n.s.	-0.11	***
BIO5	Max Temperature of Warmest Month	0.28	***	0.06	**	0.25	***
BIO6	Min Temperature of Coldest Month	0.25	***	0.01	n.s.	0.22	***
BIO7	Temperature Annual Range (BIO5-BIO6)	-0.09	***	0.04	**	-0.07	***
BIO8	Mean Temperature of Wettest Quarter	0.14	***	0.03	n.s.	0.18	***
BIO9	Mean Temperature of Driest Quarter	0.25	***	0.02	n.s.	0.22	***
BIO10	Mean Temperature of Warmest Quarter	0.29	***	0.06	**	0.25	***
BIO11	Mean Temperature of Coldest Quarter	0.27	***	0.01	n.s.	0.23	***
BIO12	Total Annual Precipitation	-0.02	**	0.07	***	-0.04	***

Supplementary Table 3 Colour coding relative to the degree of cell wall lignification (DCWL). Different hue, saturation and brightness ranges were applied to define colour in anatomical wood cross-sections.

**Colour**	**Hue**	**Saturation**	**Brightness**
red	40-189	40-255	40-255
blue	125-189	40-255	120-255
white	0-255	0-29	120-255

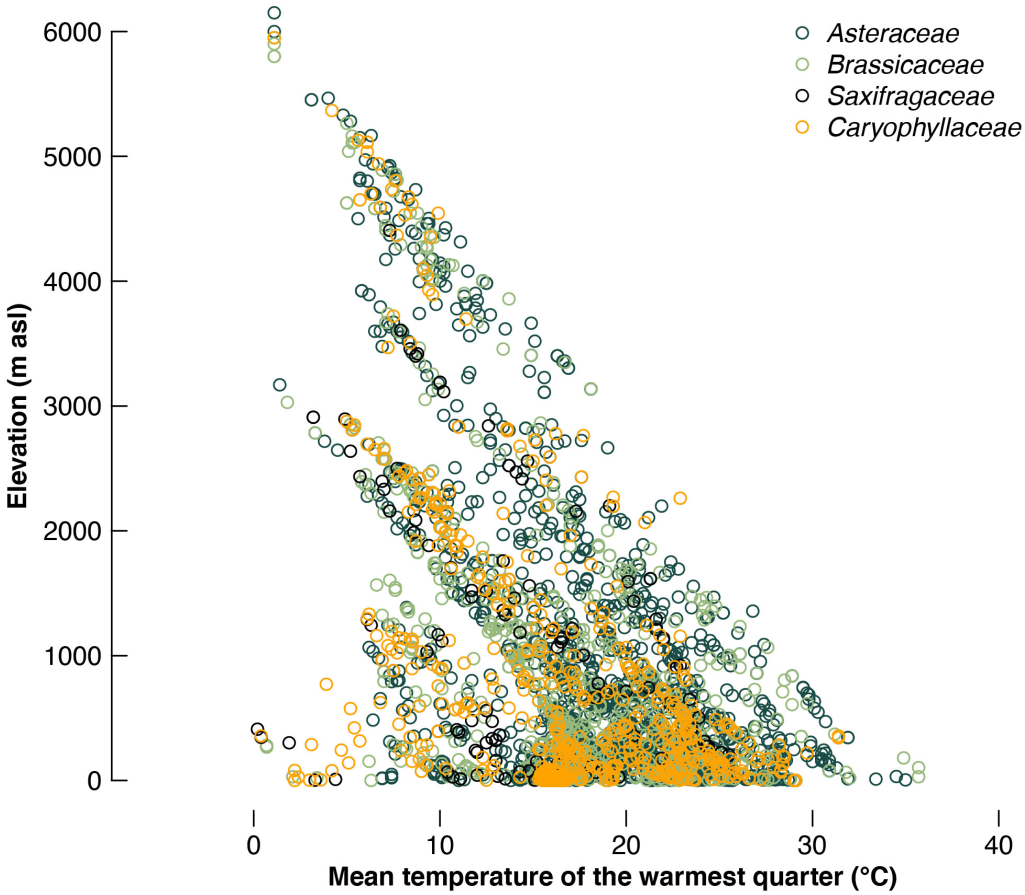

Supplementary Fig. 1 Relationship between elevational, temperature and plant families. Location of occurrence points for species belonging to the families *Asteraceae*, *Brassicaceae*, *Saxifragaceae* and *Caryophyllaceae* in relation to elevation and mean temperature of the warmest quarter.

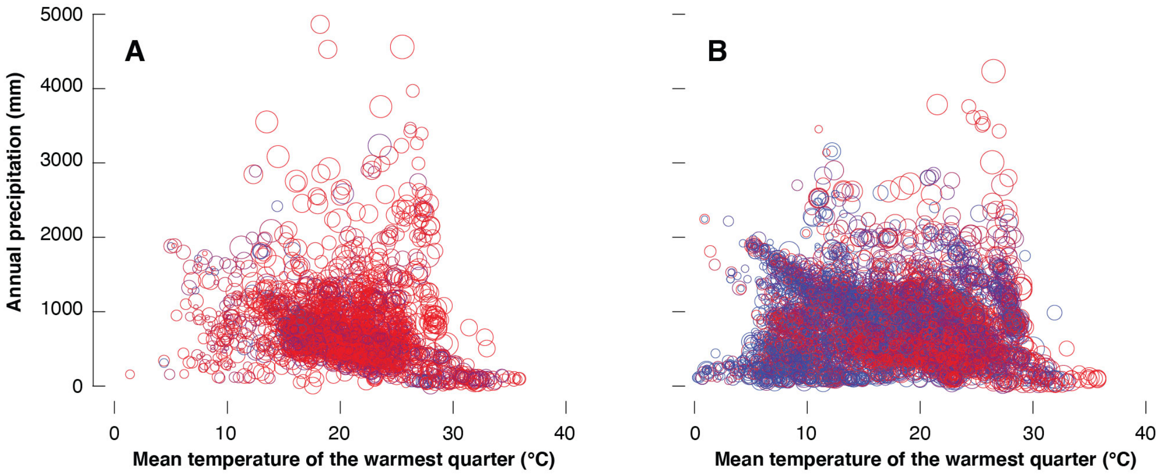

Supplementary Fig. 2 Plant cell wall lignification and climate variation. Location of (**A**) the individual tree and shrub species, as well as (**B**) the herb species occurrence points in relation to the total annual precipitation and mean temperature of the warmest quarter. Observations are color-coded from almost non-lignified (blue) to nearly fully lignified (red), and the size of the circles refers to plant height.

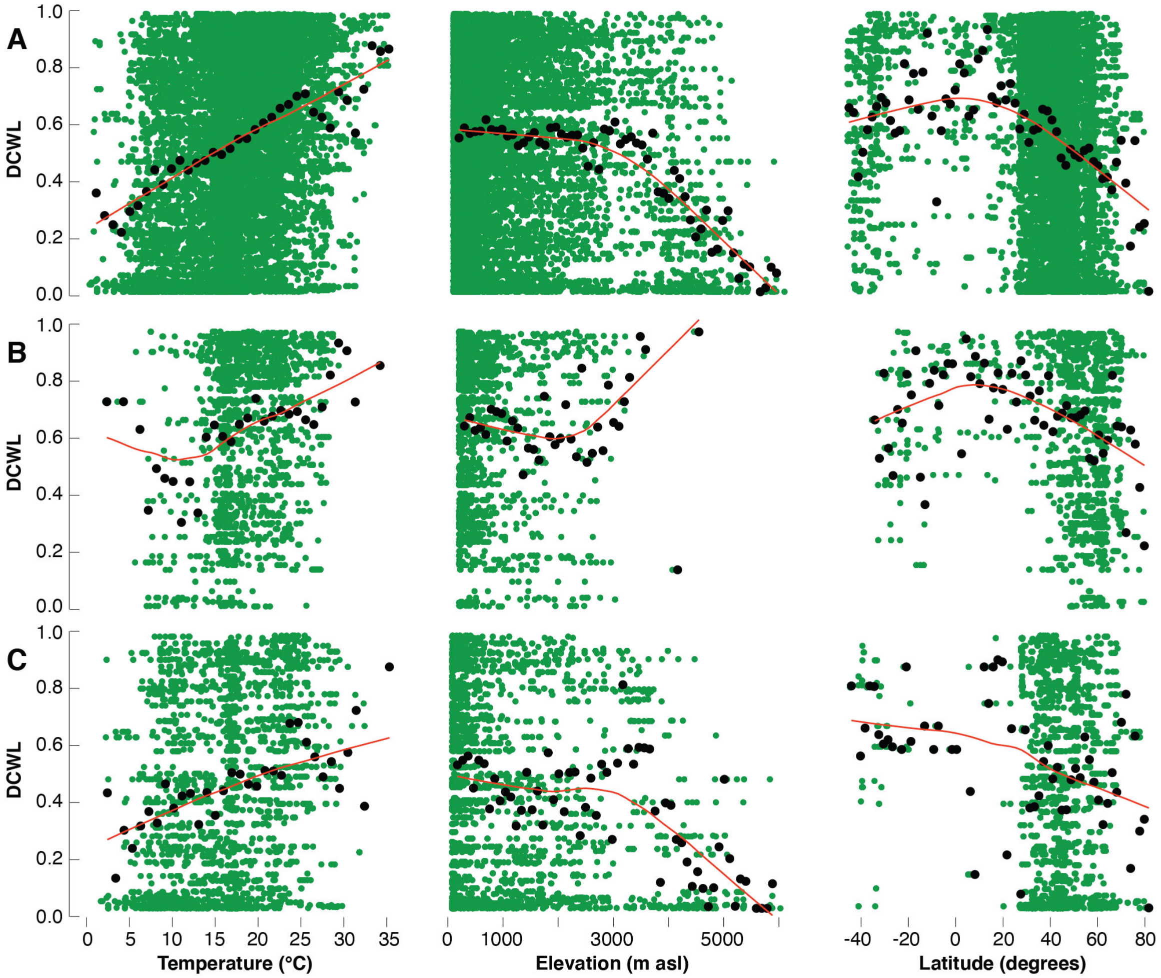

Supplementary Fig. 3 Relationship between plant cell wall lignification, temperature, elevation and latitude. Plots are calculated separately for (**A**) all samples from natural habitats, (**B**) all samples from botanic gardens, and (**C**) all herbs smaller ten cm vertical height. The green dots represent species-site combinations, while black dots are mean values of DCWL computed in each class of 1°C temperature of the warmest quarter, 100-meter elevation, and 2° latitude (from left to right). Red lines are locally weighted polynomial Loess Regressions fitted to the mean DCWL values.

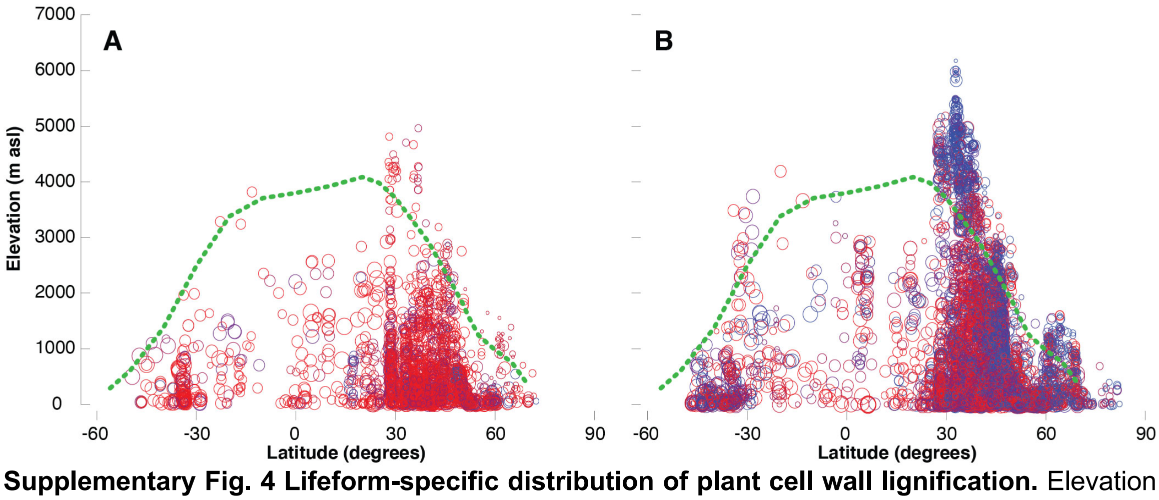

Supplementary Fig. 4 Lifeform-specific distribution of plant cell wall lignification. Elevation and latitude distribution of species-site combinations of (**A**) trees and shrubs, as well as (**B**) herbs. Colours referring to DCWL (from almost non-lignified blue to nearly fully lignified red) and dot sizes referring proportionally to plant height. The green line describes the global treeline position[48].

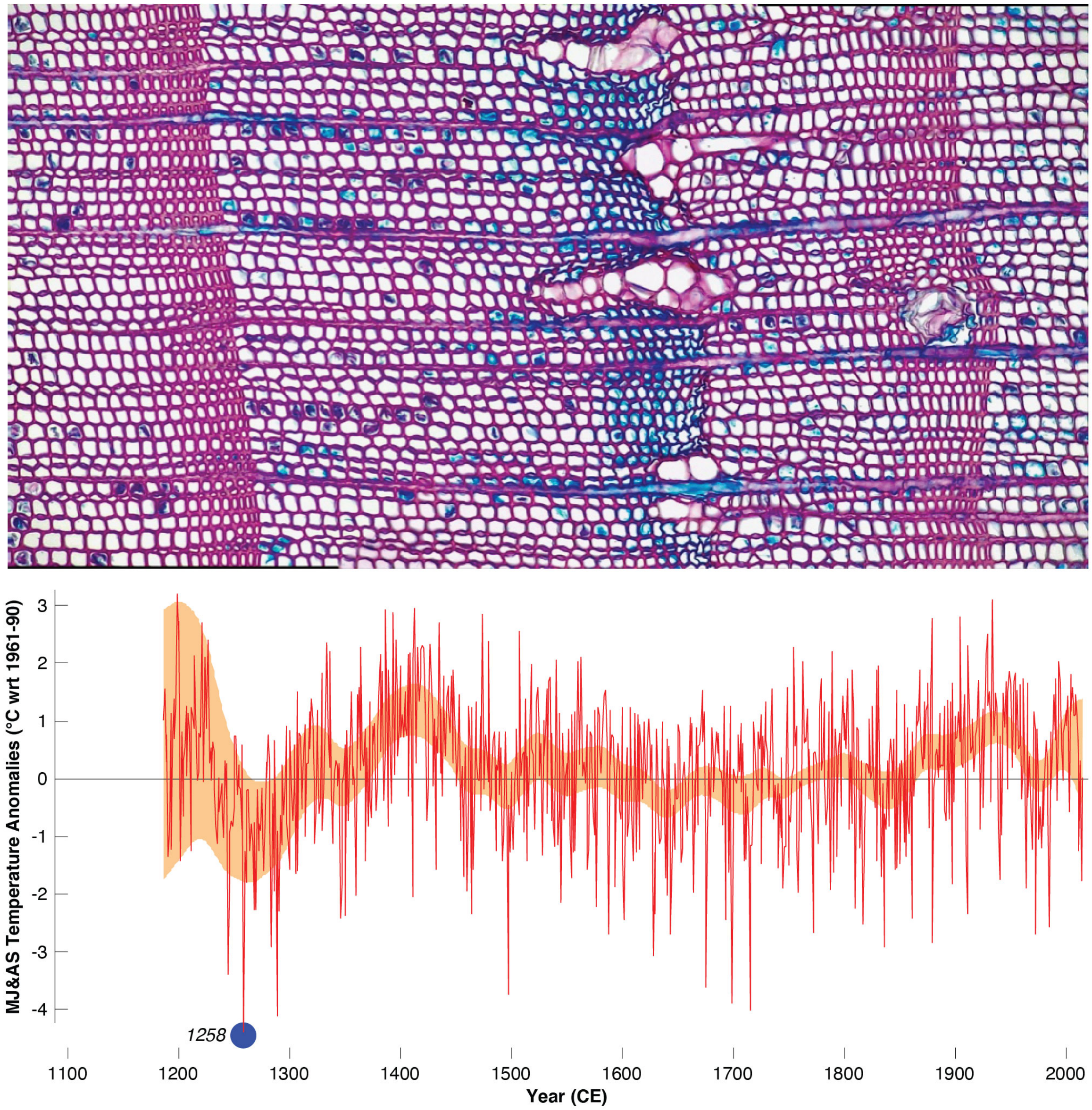

Supplementary Fig. 5 Wood anatomical 'Blue Ring' evidence from the Pyrenees. A double-stained thin section of a high-elevation (above 2,200 m asl) *Pinus uncinata* sample from the central Spanish Pyrenees that shows a so-called 'Blue Ring' and a frost damage in 1258 CE[37]. Graph shows the reconstructed May–June and August–September (MJ&AS) temperature anomalies between 1186 and 2014 CE from high-elevation, living and relict *Pinus uncinata* (Ramond ex DC.) maximum latewood density measurements from the central Spanish Pyrenees (same site of the 'Blue Ring' sample)[37], together with uncertainty ranges after 80-year low-pass filtering (orange shading). The coldest reconstructed summer is 1258 CE, following the large tropical volcanic eruptions of Samalas in 1257 CE, which is part of Rinjani volcano in Indonesia.
