# Supplementary material for: Biogeographic implication of temperature-induced plant cell wall lignification
Source: Commun Biol. 2022 Jul 29;5:767. doi: 10.1038/s42003-022-03732-y (PMC9338036; doi:10.1038/s42003-022-03732-y)
Supplement: Supplementary file 2 — Description of Additional Supplementary Files [file 42003_2022_3732_MOESM2_ESM.pdf]

Description of Additional Supplementary Files

File name: Supplementary Data 1

Description: Contains all relevant data.

File name: Supplementary Code 1

Description: Contains all relevant codes.
