# Supplementary material for: Biogeographic implication of temperature-induced plant cell wall lignification
Source: Commun Biol. 2022 Jul 29;5:767. doi: 10.1038/s42003-022-03732-y (PMC9338036; doi:10.1038/s42003-022-03732-y)
Supplement: Supplementary file 4 — Supplementary Code 1 [file 42003_2022_3732_MOESM4_ESM.docx]

Code information for

**Biogeographic implication of a thermally-constraint global distribution of plant cell wall lignification**

by Alan Crivellaro, Alma Piermattei, Jiri Dolezal, Paul Dupree and Ulf Büntgen

# start with a clean workspace

rm(list=ls())

# reset all graphic parameters

op <- par(no.readonly = TRUE)

# set working directory

setwd("/Users/Alan/Documents/PAPERS AND BOOKS/2019_Lignin Data Zuest 2016/R data in use")

# install and attach the following libraries

require(ggplot2)

require(plotly)

require(ggExtra)

require(FactoMineR)

require(plyr)

require(gridExtra)

require(grid)

require(maps)

require(plotrix)

require(vioplot)

# load data

rm(list=ls())

op <- par(no.readonly = TRUE)

Lig <- read.csv("DCWL_data.txt", sep="\t")

##############

# Data subsets

##############

Lig_TR <- Lig[Lig[,"LifeForm"] == "Trubs", ] # create subset only Trubs

Lig_HE <- Lig[Lig[,"LifeForm"] == "Herb", ] # create subset only Herbs

Lig_HE10 <- Lig_HE[Lig_HE[,"PlantH"] <= 10, ] # create subset of Herbs shorter than 10 cm

Lig_TR_HE <- Lig[Lig[,"LifeForm"] != "Climber", ] # create subset only Trubs and Herb

Lig_sp <- distinct(Lig, name, .keep_all = TRUE) # una sola specie, senza ripetizioni.

Lig_sp_TR <- Lig_sp[Lig_sp[,"LifeForm"] == "Trubs", ] # create subset only Trubs

Lig_sp_HE <- Lig_sp[Lig_sp[,"LifeForm"] == "Herb", ] # create subset only Herbs

Lig_wild <- Lig[Lig[,"Sampling_site"] == "wild", ] # create subset only samples from thw wild, not botaninc garden

Lig_botg <- Lig[Lig[,"Sampling_site"] == "Garden", ] # create subset only botaninc garden

##############

# Fig. 3 | Global dataset of plant cell wall lignification.

##############

nf <- layout(matrix(c(1,1,2,3),2,2,byrow=TRUE), widths=c(1,3), heights=c(2,1), TRUE)

map("world",

interior=FALSE, boundary=TRUE, myborder= -0.03, resolution=0.1,

ylim=c(-60, 90), col="gray30", fill=FALSE, bg="white")

points(Lig$Longitude, Lig$Latitude, col = "#58A181", cex = 0.4, pch=20)

par(new=T)

SitesMAP <- read.csv("DATASETLignin.txt", sep="\t")

points(SitesMAP$Longitude, SitesMAP$Latitude, col = "black", pch=16, cex=0.6)

legend("bottomleft", c("Occurence site", "Sampling site"), pch=c(16,16), col=c("#58A181", "black"), bty="n", cex=1)

par(mar=c(4,4,0,0))

par(las=1,bty="n", mgp = c(3, 0.6, 0))

vioplot(Lig_sp_TR$DCWL, #on unique species

Lig_sp_HE$DCWL, #on unique species

ylim=c(0,1), yaxt="r",

names=c("Trubs", "Herbs"), col=c("#2f4b7c", "#ffa600"))

title(ylab="Degree of cell wall lignification", cex.lab=0.9, font=2)

points(1, mean(Lig_sp_TR$DCWL), pch=16, cex=1, col="#d45087")

points(2, mean(Lig_sp_HE$DCWL), pch=16, cex=1, col="#d45087")

text(1.5, 1, "***", cex= 1.3)

par(mar=c(6,5,0,1.5))

par(las=1,bty="n")

boxplot((Lig_sp_TR[Lig_sp_TR$Family=="Asteraceae",])$DCWL,

(Lig_sp_HE[Lig_sp_HE$Family=="Asteraceae",])$DCWL,

(Lig_sp_TR[Lig_sp_TR$Family=="Fabaceae",])$DCWL,

(Lig_sp_HE[Lig_sp_HE$Family=="Fabaceae",])$DCWL,

(Lig_sp_TR[Lig_sp_TR$Family=="Brassicaceae",])$DCWL,

(Lig_sp_HE[Lig_sp_HE$Family=="Brassicaceae",])$DCWL,

(Lig_sp_TR[Lig_sp_TR$Family=="Lamiaceae",])$DCWL,

(Lig_sp_HE[Lig_sp_HE$Family=="Lamiaceae",])$DCWL,

(Lig_sp_TR[Lig_sp_TR$Family=="Caryophyllaceae",])$DCWL,

(Lig_sp_HE[Lig_sp_HE$Family=="Caryophyllaceae",])$DCWL,

(Lig_sp_TR[Lig_sp_TR$Family=="Plantaginaceae",])$DCWL,

(Lig_sp_HE[Lig_sp_HE$Family=="Plantaginaceae",])$DCWL,

(Lig_sp_TR[Lig_sp_TR$Family=="Apiaceae",])$DCWL,

(Lig_sp_HE[Lig_sp_HE$Family=="Apiaceae",])$DCWL,

(Lig_sp_TR[Lig_sp_TR$Family=="Rosaceae",])$DCWL,

(Lig_sp_HE[Lig_sp_HE$Family=="Rosaceae",])$DCWL,

(Lig_sp_TR[Lig_sp_TR$Family=="Orobanchaceae",])$DCWL,

(Lig_sp_HE[Lig_sp_HE$Family=="Orobanchaceae",])$DCWL,

(Lig_sp_TR[Lig_sp_TR$Family=="Boraginaceae",])$DCWL,

(Lig_sp_HE[Lig_sp_HE$Family=="Boraginaceae",])$DCWL,

(Lig_sp_TR[Lig_sp_TR$Family=="Euphorbiaceae",])$DCWL,

(Lig_sp_HE[Lig_sp_HE$Family=="Euphorbiaceae",])$DCWL,

(Lig_sp_TR[Lig_sp_TR$Family=="Amaranthaceae",])$DCWL,

(Lig_sp_HE[Lig_sp_HE$Family=="Amaranthaceae",])$DCWL,

(Lig_sp_TR[Lig_sp_TR$Family=="Ranunculaceae",])$DCWL,

(Lig_sp_HE[Lig_sp_HE$Family=="Ranunculaceae",])$DCWL,

(Lig_sp_TR[Lig_sp_TR$Family=="Polygonaceae",])$DCWL,

(Lig_sp_HE[Lig_sp_HE$Family=="Polygonaceae",])$DCWL,

(Lig_sp_TR[Lig_sp_TR$Family=="Campanulaceae",])$DCWL,

(Lig_sp_HE[Lig_sp_HE$Family=="Campanulaceae",])$DCWL,

(Lig_sp_TR[Lig_sp_TR$Family=="Scrophulariaceae",])$DCWL,

(Lig_sp_HE[Lig_sp_HE$Family=="Scrophulariaceae",])$DCWL,

(Lig_sp_TR[Lig_sp_TR$Family=="Ericaceae",])$DCWL,

(Lig_sp_HE[Lig_sp_HE$Family=="Ericaceae",])$DCWL,

ylab="DCWL", xlab="", las=1,

col=c("#2f4b7c","#ffa600"),rep(2), varwidth=TRUE,

ylim=c(-0.04,1.02), xlim=c(0,34), xaxt="n",

outpch=20, cex = 0.2)

xlabpos<-c(1.5,3.5,5.5,7.5,9.5,11.5,13.5,15.5,17.5,19.5,21.5,23.5,25.5,27.5, 29.5, 31.5, 33.5)

xlabtext<-c("Asteraceae", "Fabaceae", "Brassicaceae", "Lamiaceae", "Caryophyllaceae", "Plantaginaceae", "Apiaceae", "Rosaceae", "Orobanchaceae", "Boraginaceae", "Euphorbiaceae", "Amarantaceae", "Ranunculaceae", "Polygonaceae", "Campanulaceae", "Scrophulariaceae", "Ericaceae")

text(xlabpos, par("usr")[3] -0, srt=45, adj=1, labels=xlabtext, xpd = TRUE)

for(i in seq(0.5 , 36 , 2)){ ablineclip(v=i, y1=-0.5, y2=1, lty=1, col="gray80")} # requires plotrix

##############

# Fig. 4 | Relation between cell wall lignification and plant height.

##############

rbPal <- colorRampPalette(c('blue','red'))

Lig$Col <- rbPal(10)[as.numeric(cut(Lig$DCWL, breaks = 10))]

plot(Lig$PlantH/100, Lig$DCWL, las=1,

xlab="", ylab="",

xlim=c(0,40), ylim=c(0,1),

col=Lig$Col, bty="n", pch=20, cex=0.6, mgp = c(3, 0.6, 0))

mtext(text = "Plant height (m)", side = 1, line = 2, cex=1, font=2)

mtext(text = "Degree of cell wall lignification (DCWL)", side = 2, line = 2.2, cex=1, font=2)

##############

# Fig. 5 | The influence of temperature, elevation and latitude on plant cell wall lignification.

##############

nf <- layout(matrix(c(1,2,3),1,3,byrow=TRUE), widths=c(1,1), heights=c(1,1), TRUE)

# A

par(mar=c(4,4,1,0), lwd=2)

plot(Lig$BIO10/10, Lig$DCWL, las=1,

xlab="", ylab="", cex.lab=1,

xlim=c(0,36), ylim=c(0,1),

col="forestgreen", bty="n", pch=20, cex=0.5, mgp = c(3, 0.6, 0))

mtext(text = "Mean temperature of warmest quarter (\u00B0C)", side = 1, line = 1.8, cex=0.5, font=2)

mtext(text = "Degree of cell wall lignification (DCWL)", side = 2, line = 2.4, cex=0.5, font=2)

par(new=T)

Lig$BIO10Cut <- cut(Lig$BIO10/10, seq(0,36,1), labels = c(1:36))

agg_BIO10 <- aggregate(Lig$DCWL, by=list(Lig$BIO10Cut), mean)

scatter.smooth(agg_BIO10$Group.1, agg_BIO10$x,

main="", ylab="", xlab="", las=1, bty="n",

xlim=c(0,36), ylim=c(0,1), col="black", pch=16, axes=FALSE)

# B

par(mar=c(4,3,1,0), lwd=2)

plot(Lig$Elevation, Lig$DCWL, las=1,

xlab="", ylab="", cex.lab=1,

xlim=c(0,6000), ylim=c(0,1),

col="forestgreen", bty="n", pch=20, cex=0.5, mgp = c(3, 0.6, 0))

mtext(text = "Elevation (m asl)", side = 1, line = 1.8, cex=0.5, font=2)

par(new=T)

Lig$ElevationCut <- cut(Lig$Elevation, seq(0,6000,100), labels = c(1:60))

agg_elev <- aggregate(Lig$DCWL, by=list(Lig$ElevationCut),mean)

scatter.smooth(agg_elev$Group.1, agg_elev$x,

main="", ylab="", xlab="", las=1, bty="n",

xlim=c(0,60), ylim=c(0,1),col="black",pch=16, axes=FALSE)

# C

par(mar=c(4,3,1,0), lwd=2)

plot(Lig$Latitude, Lig$DCWL, las=1,

xlab="", ylab="", cex.lab=1,

xlim=c(-50,90), ylim=c(0,1),

col="forestgreen", bty="n", pch=20, cex=0.5, mgp = c(3, 0.6, 0))

mtext(text = "Latitude (Degrees)", side = 1, line = 1.8, cex=0.5, font=2)

par(new=T)

Lig$latitudeCut <- cut(Lig$Latitude, seq(-50,90,2), labels = c(1:70))

agg_lat <- aggregate(Lig$DCWL, by=list(Lig$latitudeCut), mean)

scatter.smooth(agg_lat$Group.1, agg_lat$x,

main="", ylab="", xlab="", las=1, bty="n",

xlim=c(0,70), ylim=c(0,1), col="black", pch=16, axes=FALSE)

##############

# Fig. 6 | Global distribution of plant cell wall lignification.

##############

x <- c(70,65,60,55,52,50,40,30,25,20,10,0,-10,-20,-30,-40,-50,-56) #treeline latitudes

y <- c(300,850,1000,1100,1500,2000,2900,3700,4000,4100,3900,3800,3700,3400,2500,1350,600,300) #treeline altitudes

smoothingSpline = smooth.spline(x, y, spar=0.3)

rbPal <- colorRampPalette(c('blue','red'))

Lig$Col <- rbPal(10)[as.numeric(cut(Lig$DCWL, breaks = 10))]

plot(Lig$Latitude, Lig$Elevation,

xlab="", ylab="", xaxt="n", yaxt="n",

xlim=c(-60,90), ylim=c(0,7000), pch=20,

col = Lig$Col, bty="n", mgp = c(3, 0.6, 0))#plot(x,y, xlim=c(-60,90), ylim=c(0,7000), pch="",

axis(1, xaxp = c(-60, 90, 5), mgp = c(3, 0.6, 0))

axis(2, xaxp = c(0, 7000, 8), las=1, mgp = c(3, 0.6, 0))

mtext(text = "Latitude (Degree)", side = 1, line = 1.8, cex=1, font=2)

mtext(text = "Elevation (m asl)", side = 2, line = 2.8, cex=1, font=2)

lines(smoothingSpline, lwd=3, col="green")

par(new=T)

par(mar=c(4,4,1,0), lwd=2)

text(-64, 7200, "A", cex=1.1, font=2)

### inlet treeline graph with plot only extremes

Lig_extr <- Lig[Lig[,"DCWL"] >= 0.98 | Lig[,"DCWL"] <= 0.02, ] # uppermost and lowermost quartiles

#rbPal <- colorRampPalette(c('blue','red'))

Lig_extr$Col <- rbPal(10)[as.numeric(cut(Lig_extr$DCWL, breaks = 10))]

# library(oce)

par(las=1)

plotInset(-60, 4100, 0, 7050,

expr=

plot(Lig_extr$Latitude, Lig_extr$Elevation,

xlab="", ylab="", main="",

las=1, col = Lig_extr$Col,

xlim=c(-60,90), ylim=c(0,6200),

yaxt="n", xaxt="n", cex=0.4, pch=16, fg="grey"))

lines(smoothingSpline, lwd=1, col="black")

text(-58, 6700, "B", cex=1.1, font=2)

##############

# Extended Data Table 2 | Relationships between the Degree of Cell Wall Lignification (DCWL) and bioclimatic variables.

##############

# all

for(i in 10:21) {

print(cor(Lig_TR_HE[,"DCWL"],Lig_TR_HE[,i], use="complete.obs"))

}

for(i in 10:21) {

print(cor.test(Lig_TR_HE[,"DCWL"],Lig_TR_HE[,i], use="complete.obs")$p.value)

}

#Trub

for(i in 10:21) {

print(cor(Lig_TR[,"DCWL"],Lig_TR[,i], use="complete.obs"))

}

for(i in 10:21) {

print(cor.test(Lig_TR[,"DCWL"],Lig_TR[,i], use="complete.obs")$p.value)

}

# Herb

for(i in 10:21) {

print(cor(Lig_HE[,"DCWL"],Lig_HE[,i], use="complete.obs"))

}

for(i in 10:21) {

print(cor.test(Lig_HE[,"DCWL"],Lig_HE[,i], use="complete.obs")$p.value)

}

##############

# Extended Data Fig. 1 | Relationship between elevational, temperature and plant families.

##############

Ast <- (Lig[Lig$Family=="Asteraceae",])

Bra <- (Lig[Lig$Family=="Brassicaceae",])

Sax <- (Lig[Lig$Family=="Saxifragaceae",])

Car <- (Lig[Lig$Family=="Caryophyllaceae",])

plot(Ast$BIO10/10, Ast$Elevation,

bty="n",

xlim=c(-5,40), ylim=c(0,6000),

xlab="", ylab="", las=1, mgp = c(3, 0.6, 0),

pch=1, col="#184d47")

mtext(text = "Mean temperature of the warmest quarter (\u00B0C)", side = 1, line = 1.8, cex=1, font=2)

mtext(text = "Elevation (m asl)", side = 2, line = 2.8, cex=1, font=2)

par(new=T)

plot(Bra$BIO10/10, Bra$Elevation,

bty="n",

xlim=c(-5,40), ylim=c(0,6000),

xlab="", ylab="", las=1, mgp = c(3, 0.6, 0),

pch=1, col="#96bb7c", axes=FALSE)

par(new=T)

plot(Sax$BIO10/10, Sax$Elevation,

bty="n",

xlim=c(-5,40), ylim=c(0,6000),

xlab="", ylab="", las=1, mgp = c(3, 0.6, 0),

pch=1, col="black", axes=FALSE)

par(new=T)

plot(Car$BIO10/10, Car$Elevation,

bty="n",

xlim=c(-5,40), ylim=c(0,6000),

xlab="", ylab="", las=1, mgp = c(3, 0.6, 0),

pch=1, col="orange", axes=FALSE)

legend("topright",

c("Asteraceae", "Brassicaceae", "Saxifragaceae", "Caryophyllaceae"),

pch=c(1,1,1,1),

col=c("#184d47", "#96bb7c", "black", "orange"),

bty="n", cex=1)

##############

# Extended Data Fig. 2 | Plant cell wall lignification and climate variation.

##############

rbPal <- colorRampPalette(c('blue','red'))

Lig_TR$Col <- rbPal(10)[as.numeric(cut(Lig_TR$DCWL, breaks = 10))]

nf <- layout(matrix(c(1,2),1,2,byrow=TRUE), widths=c(4,4), heights=c(4,4), TRUE)

symbols(Lig_TR$BIO10/10, Lig_TR$BIO12,

circles=log(Lig_TR$PlantH), inches=1/20, bty="n",

bg=Lig_TR$Col, fg=NULL,

xlim=c(-5,40), ylim=c(0,5000),

xlab="", ylab="", las=1, mgp = c(3, 0.6, 0))

mtext(text = "Mean temperature of the warmest quarter (\u00B0C)", side = 1, line = 1.8, cex=1, font=2)

mtext(text = "Total annual precipitation (mm)", side = 2, line = 2.8, cex=1, font=2)

rbPal <- colorRampPalette(c('blue','red'))

Lig_HE$Col <- rbPal(10)[as.numeric(cut(Lig_HE$DCWL, breaks = 10))]

symbols(Lig_HE$BIO10/10, Lig_HE$BIO12,

circles=log(Lig_HE$PlantH), inches=1/20, bty="n",

bg=Lig_HE$Col, fg=NULL,

xlim=c(-5,40), ylim=c(0,5000),

xlab="", ylab="", las=1, mgp = c(3, 0.6, 0))

mtext(text = "Mean temperature of the warmest quarter (\u00B0C)", side = 1, line = 1.8, cex=1, font=2)

mtext(text = "Total annual precipitation (mm)", side = 2, line = 2.8, cex=1, font=2)

##############

# Extended Data Fig. 3 | Relationship between plant cell wall lignification, temperature, elevation and latitude.

##############

nf <- layout(matrix(c(1,2,3),1,3,byrow=TRUE), widths=c(1,1), heights=c(1,1), TRUE)

###### wild sampling

# A # Lig vs temp

par(mar=c(4,4,1,0), lwd=2)

plot(Lig_wild$BIO10/10, Lig_wild$DCWL, las=1,

xlab="", ylab="", cex.lab=1,

xlim=c(0,36), ylim=c(0,1),

col="forestgreen", bty="n", pch=20, mgp = c(3, 0.6, 0))

mtext(text = "Mean temperature of warmest quarter (\u00B0C)", side = 1, line = 1.8, cex=0.5, font=2)

mtext(text = "Degree of cell wall lignification (DCWL)", side = 2, line = 2.4, cex=0.5, font=2)

par(new=T)

Lig_wild$BIO10Cut <- cut(Lig_wild$BIO10/10, seq(0,36,1), labels = c(1:36))

agg_BIO10 <- aggregate(Lig_wild$DCWL, by=list(Lig_wild$BIO10Cut), mean)

scatter.smooth(agg_BIO10$Group.1, agg_BIO10$x,

main="", ylab="", xlab="", las=1, bty="n",

xlim=c(0,36), ylim=c(0,1), col="black", pch=16, axes=FALSE)

# B # Lig vs Elevation

par(mar=c(4,3,1,0), lwd=2)

plot(Lig_wild$Elevation, Lig_wild$DCWL, las=1,

xlab="", ylab="", cex.lab=1,

xlim=c(0,6000), ylim=c(0,1),

col="forestgreen", bty="n", pch=20, mgp = c(3, 0.6, 0))

mtext(text = "Elevation (m asl)", side = 1, line = 1.8, cex=0.5, font=2)

par(new=T)

Lig_wild$ElevationCut <- cut(Lig_wild$Elevation, seq(0,6000,100), labels = c(1:60))

agg_elev <- aggregate(Lig_wild$DCWL, by=list(Lig_wild$ElevationCut),mean)

scatter.smooth(agg_elev$Group.1, agg_elev$x,

main="", ylab="", xlab="", las=1, bty="n",

xlim=c(0,60), ylim=c(0,1),col="black",pch=16, axes=FALSE)

# C # Lig vs latitude

par(mar=c(4,3,1,0), lwd=2)

plot(Lig_wild$Latitude, Lig_wild$DCWL, las=1,

xlab="", ylab="", cex.lab=1,

xlim=c(-50,90), ylim=c(0,1),

col="forestgreen", bty="n", pch=20, mgp = c(3, 0.6, 0))

mtext(text = "Latitude (Degrees)", side = 1, line = 1.8, cex=0.5, font=2)

par(new=T)

Lig_wild$latitudeCut <- cut(Lig_wild$Latitude, seq(-50,90,2), labels = c(1:70))

agg_lat <- aggregate(Lig_wild$DCWL, by=list(Lig_wild$latitudeCut), mean)

scatter.smooth(agg_lat$Group.1, agg_lat$x,

main="", ylab="", xlab="", las=1, bty="n",

xlim=c(0,70), ylim=c(0,1), col="black", pch=16, axes=FALSE)

# botanic garden samples

##############

nf <- layout(matrix(c(1,2,3),1,3,byrow=TRUE), widths=c(1,1), heights=c(1,1), TRUE)

# A

par(mar=c(4,4,1,0), lwd=2)

plot(Lig_botg$BIO10/10, Lig_botg$DCWL, las=1,

xlab="", ylab="", cex.lab=1,

xlim=c(0,36), ylim=c(0,1),

col="forestgreen", bty="n", pch=20, mgp = c(3, 0.6, 0))

mtext(text = "Mean temperature of warmest quarter (\u00B0C)", side = 1, line = 1.8, cex=0.5, font=2)

mtext(text = "Degree of cell wall lignification (DCWL)", side = 2, line = 2.4, cex=0.5, font=2)

par(new=T)

Lig_botg$BIO10Cut <- cut(Lig_botg$BIO10/10, seq(0,36,1), labels = c(1:36))

agg_BIO10 <- aggregate(Lig_botg$DCWL, by=list(Lig_botg$BIO10Cut), mean)

scatter.smooth(agg_BIO10$Group.1, agg_BIO10$x,

main="", ylab="", xlab="", las=1, bty="n",

xlim=c(0,36), ylim=c(0,1), col="black", pch=16, axes=FALSE)

# B # Lig vs Elevation

par(mar=c(4,3,1,0), lwd=2)

plot(Lig_botg$Elevation, Lig_botg$DCWL, las=1,

xlab="", ylab="", cex.lab=1,

xlim=c(0,6000), ylim=c(0,1),

col="forestgreen", bty="n", pch=20, mgp = c(3, 0.6, 0))

mtext(text = "Elevation (m asl)", side = 1, line = 1.8, cex=0.5, font=2)

par(new=T)

Lig_botg$ElevationCut <- cut(Lig_botg$Elevation, seq(0,6000,100), labels = c(1:60))

agg_elev <- aggregate(Lig_botg$DCWL, by=list(Lig_botg$ElevationCut),mean)

scatter.smooth(agg_elev$Group.1, agg_elev$x,

main="", ylab="", xlab="", las=1, bty="n",

xlim=c(0,60), ylim=c(0,1),col="black",pch=16, axes=FALSE)

# C # Lig vs latitude

par(mar=c(4,3,1,0), lwd=2)

plot(Lig_botg$Latitude, Lig_botg$DCWL, las=1,

xlab="", ylab="", cex.lab=1,

xlim=c(-50,90), ylim=c(0,1),

col="forestgreen", bty="n", pch=20, mgp = c(3, 0.6, 0))

mtext(text = "Latitude (Degrees)", side = 1, line = 1.8, cex=0.5, font=2)

par(new=T)

Lig_botg$latitudeCut <- cut(Lig_botg$Latitude, seq(-50,90,2), labels = c(1:70))

agg_lat <- aggregate(Lig_botg$DCWL, by=list(Lig_botg$latitudeCut), mean)

scatter.smooth(agg_lat$Group.1, agg_lat$x,

main="", ylab="", xlab="", las=1, bty="n",

xlim=c(0,70), ylim=c(0,1), col="black", pch=16, axes=FALSE)

# Herbs with plant heigh shorter that 10 cm

##############

nf <- layout(matrix(c(1,2,3),1,3,byrow=TRUE), widths=c(1,1), heights=c(1,1), TRUE)

# A

par(mar=c(4,4,1,0), lwd=2)

plot(Lig_HE10$BIO10/10, Lig_HE10$DCWL, las=1,

xlab="", ylab="", cex.lab=1,

xlim=c(0,36), ylim=c(0,1),

col="forestgreen", bty="n", pch=20, mgp = c(3, 0.6, 0))

mtext(text = "Mean temperature of warmest quarter (\u00B0C)", side = 1, line = 1.8, cex=0.5, font=2)

mtext(text = "Degree of cell wall lignification (DCWL)", side = 2, line = 2.4, cex=0.5, font=2)

par(new=T)

Lig_HE10$BIO10Cut <- cut(Lig_HE10$BIO10/10, seq(0,36,1), labels = c(1:36))

agg_BIO10 <- aggregate(Lig_HE10$DCWL, by=list(Lig_HE10$BIO10Cut), mean)

scatter.smooth(agg_BIO10$Group.1, agg_BIO10$x,

main="", ylab="", xlab="", las=1, bty="n",

xlim=c(0,36), ylim=c(0,1), col="black", pch=16, axes=FALSE)

# B # Lig vs Elevation

par(mar=c(4,3,1,0), lwd=2)

plot(Lig_HE10$Elevation, Lig_HE10$DCWL, las=1,

xlab="", ylab="", cex.lab=1,

xlim=c(0,6000), ylim=c(0,1),

col="forestgreen", bty="n", pch=20, mgp = c(3, 0.6, 0))

mtext(text = "Elevation (m asl)", side = 1, line = 1.8, cex=0.5, font=2)

par(new=T)

Lig_HE10$ElevationCut <- cut(Lig_HE10$Elevation, seq(0,6000,100), labels = c(1:60))

agg_elev <- aggregate(Lig_HE10$DCWL, by=list(Lig_HE10$ElevationCut),mean)

scatter.smooth(agg_elev$Group.1, agg_elev$x,

main="", ylab="", xlab="", las=1, bty="n",

xlim=c(0,60), ylim=c(0,1),col="black",pch=16, axes=FALSE)

# C # Lig vs latitude

par(mar=c(4,3,1,0), lwd=2)

plot(Lig_HE10$Latitude, Lig_HE10$DCWL, las=1,

xlab="", ylab="", cex.lab=1,

xlim=c(-50,90), ylim=c(0,1),

col="forestgreen", bty="n", pch=20, mgp = c(3, 0.6, 0))

mtext(text = "Latitude (Degrees)", side = 1, line = 1.8, cex=0.5, font=2)

par(new=T)

Lig_HE10$latitudeCut <- cut(Lig_HE10$Latitude, seq(-50,90,2), labels = c(1:70))

agg_lat <- aggregate(Lig_HE10$DCWL, by=list(Lig_HE10$latitudeCut), mean)

scatter.smooth(agg_lat$Group.1, agg_lat$x,

main="", ylab="", xlab="", las=1, bty="n",

xlim=c(0,70), ylim=c(0,1), col="black", pch=16, axes=FALSE)

##############

# Extended Data Fig. 4 | Lifeform-specific distribution of plant cell wall lignification.

##############

x <- c(70,65,60,55,52,50,40,30,25,20,10,0,-10,-20,-30,-40,-50,-56) #treeline latitudes

y <- c(300,850,1000,1100,1500,2000,2900,3700,4000,4100,3900,3800,3700,3400,2500,1350,600,300) #treeline altitudes

smoothingSpline = smooth.spline(x, y, spar=0.3)

nf <- layout(matrix(c(1,2),1,2,byrow=TRUE), widths=c(4,4), heights=c(4,4), TRUE)

rbPal <- colorRampPalette(c('blue','red'))

Lig_TR$Col <- rbPal(10)[as.numeric(cut(Lig_TR$DCWL, breaks = 10))]

plot(x,y, xlim=c(-60,90), ylim=c(0,7000), pch="",

xlab="", ylab="", bty="n", mgp = c(3, 0.6, 0), axes=F)

axis(1, xaxp = c(-60, 90, 5), mgp = c(3, 0.6, 0))

axis(2, xaxp = c(0, 7000, 8), las=1, mgp = c(3, 0.6, 0))

mtext(text = "Latitude (Degree)", side = 1, line = 1.8, cex=1, font=2)

mtext(text = "Elevation (m asl)", side = 2, line = 2.8, cex=1, font=2)

par(new=T)

symbols(Lig_TR$Latitude, Lig_TR$Elevation, xlab="", ylab="", las=1,

bg = Lig_TR$Col, fg=NULL,

xlim=c(-60,90), ylim=c(0,7000), pch=1,

circles=log(Lig_TR$PlantH), inches=1/30, bty="n", xaxt="n", yaxt="n")

lines(smoothingSpline, lwd=3, col="black")

Lig_HE$Col <- rbPal(10)[as.numeric(cut(Lig_HE$DCWL, breaks = 10))]

plot(x,y, xlim=c(-60,90), ylim=c(0,7000), pch="",

xlab="", ylab="", bty="n", mgp = c(3, 0.6, 0), axes=F)

axis(1, xaxp = c(-60, 90, 5), mgp = c(3, 0.6, 0))

axis(2, xaxp = c(0, 7000, 8), las=1, mgp = c(3, 0.6, 0))

mtext(text = "Latitude (Degree)", side = 1, line = 1.8, cex=1, font=2)

mtext(text = "Elevation (m asl)", side = 2, line = 2.8, cex=1, font=2)

par(new=T)

symbols(Lig_HE$Latitude, Lig_HE$Elevation, xlab="", ylab="", las=1,

bg = Lig_HE$Col, fg=NULL,

xlim=c(-60,90), ylim=c(0,7000), pch=1,

circles=log(Lig_HE$PlantH), inches=1/30, bty="n", xaxt="n", yaxt="n")

lines(smoothingSpline, lwd=3, col="black")
