# Supplementary material for: Biogeographic implication of temperature-induced plant cell wall lignification
Source: Commun Biol. 2022 Jul 29;5:767. doi: 10.1038/s42003-022-03732-y (PMC9338036; doi:10.1038/s42003-022-03732-y)
Supplement: Supplementary file 5 — Reporting Summary [file 42003_2022_3732_MOESM5_ESM.pdf]

nature portfolio

Corresponding author(s): Ulf Buentgen
Last updated by author(s): Jun 24, 2022

Reporting Summary

Nature Portfolio wishes to improve the reproducibility of the work that we publish. This form provides structure for consistency and transparency in reporting. For further information on Nature Portfolio policies, see our Editorial Policies and the Editorial Policy Checklist.

Statistics

For all statistical analyses, confirm that the following items are present in the figure legend, table legend, main text, or Methods section.

n/a	Confirmed	
☐	☒	The exact sample size (*n*) for each experimental group/condition, given as a discrete number and unit of measurement
☐	☒	A statement on whether measurements were taken from distinct samples or whether the same sample was measured repeatedly
☐	☒	The statistical test(s) used AND whether they are one- or two-sided<br>*Only common tests should be described solely by name; describe more complex techniques in the Methods section.*
☒	☐	A description of all covariates tested
☒	☐	A description of any assumptions or corrections, such as tests of normality and adjustment for multiple comparisons
☐	☒	A full description of the statistical parameters including central tendency (e.g. means) or other basic estimates (e.g. regression coefficient) AND variation (e.g. standard deviation) or associated estimates of uncertainty (e.g. confidence intervals)
☒	☐	For null hypothesis testing, the test statistic (e.g. *F*, *t*, *r*) with confidence intervals, effect sizes, degrees of freedom and *P* value noted<br>*Give P values as exact values whenever suitable.*
☒	☐	For Bayesian analysis, information on the choice of priors and Markov chain Monte Carlo settings
☒	☐	For hierarchical and complex designs, identification of the appropriate level for tests and full reporting of outcomes
☐	☒	Estimates of effect sizes (e.g. Cohen's *d*, Pearson's *r*), indicating how they were calculated

Our web collection on statistics for biologists contains articles on many of the points above.

Software and code

Policy information about availability of computer code

Data collection	Species collection was carried out over the last 30 years from natural and undisturbed sites, as well as from botanical gardens and cultivated lands, mostly across the Northern Hemisphere. At each sampling site, we selected adult individuals that appeared undamaged and healthy. During harvest, we sampled one adult individual per species that was usually the tallest individual of that species at that site and then recorded plant height of that individual, as well as elevation and coordinates of the sites (from a GPS device) and sites name (from the nearest geographic feature). Small plants were dug out from the ground to expose the root collar, whereas for shrubs and trees, a stem disk was cut with a saw, or a wood core was extracted with an increment borer (from the main upright stem in multi-stem shrubs).
Data analysis	All relevant codes are included in the supplementary information files.

For manuscripts utilizing custom algorithms or software that are central to the research but not yet described in published literature, software must be made available to editors and reviewers. We strongly encourage code deposition in a community repository (e.g. GitHub). See the Nature Portfolio guidelines for submitting code & software for further information.

Data

Policy information about availability of data

All manuscripts must include a data availability statement. This statement should provide the following information, where applicable:

- Accession codes, unique identifiers, or web links for publicly available datasets
- A description of any restrictions on data availability
- For clinical datasets or third party data, please ensure that the statement adheres to our policy

All relevant data are included in the supplementary information files.

Field-specific reporting

Please select the one below that is the best fit for your research. If you are not sure, read the appropriate sections before making your selection.

☐ Life sciences ☐ Behavioural & social sciences ☒ Ecological, evolutionary & environmental sciences

For a reference copy of the document with all sections, see nature.com/documents/nr-reporting-summary-flat.pdf

Ecological, evolutionary & environmental sciences study design

All studies must disclose on these points even when the disclosure is negative.

Study description	We apply wood anatomical staining techniques to develop an index of the degree of cell wall lignification (DCWL) to microscopically quantify the proportion of lignified secondary cell walls in the stems of 1,770 plant species from 198 disjunct sampling sites on six continents.
Research sample	A total of 1,770 different woody plant species from 198 disjunct sampling sites.
Sampling strategy	Species collection was carried out over the last 30 years from natural and undisturbed sites, as well as from botanical gardens and cultivated lands, mostly across the Northern Hemisphere. At each sampling site, we selected adult individuals that appeared undamaged and healthy. During harvest, we sampled one adult individual per species that was usually the tallest individual of that species at that site and then recorded plant height of that individual, as well as elevation and coordinates of the sites (from a GPS device) and sites name (from the nearest geographic feature). Small plants were dug out from the ground to expose the root collar, whereas for shrubs and trees, a stem disk was cut with a saw, or a wood core was extracted with an increment borer (from the main upright stem in multi-stem shrubs). The sampled portions of stem and cores were stored in sealed plastic bags to which we added several drops of 40% ethanol and kept it at 3–4°C until the samples were sectioned.
Data collection	Data were collected over the last 30 years.
Timing and spatial scale	Data were collected over the last 30 years.
Data exclusions	No data were excluded.
Reproducibility	A detailed description of all data used and methods applied is provided in the extensive methods section of the submitted manuscript.
Randomization	Data were split into plant lifeforms.
Blinding	No blinding was used.

Did the study involve field work? ☒ Yes ☐ No

Field work, collection and transport

Field conditions	Data were collected over the last 30 years.
Location	Stem sections of 1,770 dicotyledonous angiosperm species from six continents.
Access & import/export	All data were collected, transported and analyzed following national and international laws.
Disturbance	Our sampling did not cause any disturbance.

Reporting for specific materials, systems and methods

We require information from authors about some types of materials, experimental systems and methods used in many studies. Here, indicate whether each material, system or method listed is relevant to your study. If you are not sure if a list item applies to your research, read the appropriate section before selecting a response.

Materials & experimental systems

n/a	Involved in the study
☒	☐ Antibodies
☒	☐ Eukaryotic cell lines
☒	☐ Palaeontology and archaeology
☒	☐ Animals and other organisms
☒	☐ Human research participants
☒	☐ Clinical data
☒	☐ Dual use research of concern

Methods

n/a	Involved in the study
☒	☐ ChIP-seq
☒	☐ Flow cytometry
☒	☐ MRI-based neuroimaging
